# Comparative Analysis of Machine and Deep Learning Algorithms for Bragg Peak Estimation in Polymeric Materials for Tissue-Sparing Radiotherapy

**DOI:** 10.3390/polym17152068

**Published:** 2025-07-29

**Authors:** Koray Acici

**Affiliations:** 1 Department of Artificial Intelligence and Data Engineering, Ankara University, Ankara 06830, Türkiye; kacici@ankara.edu.tr; 2 Institute of Artificial Intelligence, Ankara University, Ankara 06560, Türkiye

**Keywords:** polymers, Bragg peak, proton therapy, machine learning, deep learning, artificial intelligence, regression

## Abstract

Proton therapy has emerged as a highly precise and tissue-sparing radiotherapy technique, capitalizing on the unique energy deposition pattern of protons characterized by the Bragg peak. Ensuring treatment accuracy relies on calibration phantoms, often composed of tissue-equivalent polymeric materials. This study investigates the dosimetric behavior of four commonly used polymers—Parylene, Epoxy, Lexan, and Mylar—by analyzing their linear energy transfer (LET) values and Bragg curve characteristics across various proton energies. Experimental LET data were collected and used to train and evaluate the predictive power for Bragg peak of multiple artificial intelligence models, including kNN, SVR, MLP, RF, LWRF, XGBoost, 1D-CNN, LSTM, and BiLSTM. These algorithms were optimized using 10-fold cross-validation and assessed through statistical error and performance metrics including *MAE*, *RAE*, *RMSE*, *RRSE*, *CC*, and *R*^2^. Results demonstrate that certain AI models, particularly RF and LWRF, accurately (in terms of all evaluation metrics) predict Bragg peaks in Epoxy polymers, reducing the reliance on costly and time-consuming simulations. In terms of *CC* and *R*^2^ metrics, the LWRF model demonstrated superior performance, achieving scores of 0.9969 and 0.9938, respectively. However, when evaluated against *MAE*, *RMSE*, *RAE*, and *RRSE* metrics, the RF model emerged as the top performer, yielding values of 12.3161, 15.8223, 10.3536, and 11.4389, in the same order. Additionally, the SVR model achieved the highest number of statistically significant differences when compared pairwise with the other eight models, showing significance against six of them. The findings support the use of AI as a robust tool for designing reliable calibration phantoms and optimizing proton therapy planning. This integrative approach enhances the synergy between materials science, medical physics, and data-driven modeling in advanced radiotherapy systems.

## 1. Introduction

Proton therapy, a form of external beam radiation therapy, has emerged as a highly effective and targeted modality for the treatment of various types of cancer, including pediatric tumors, head and neck cancers, and ocular melanomas [1,2]. Its distinct advantage lies in the Bragg peak phenomenon, where the proton beam deposits the majority of its energy at a specific depth, minimizing radiation exposure to surrounding healthy tissues [3,4]. However, to harness this precision, accurate calibration and verification of treatment planning systems are essential [5]. In this context, calibration phantoms—synthetic materials that mimic human tissue—play a vital role in validating dose distributions and beam range predictions [6]. Among these, polymer-based phantoms have gained considerable attention due to their customizable radiological properties, ease of fabrication, and tissue equivalency [7,8].

Polymers such as Parylene, Bisphenol-A-based Epoxy (DGBEA—Diglycidyl Ether of Bisphenol A, Epoxy), polycarbonate (Lexan), and polyethylene terephthalate (Mylar) have been widely used in phantom construction owing to their favorable mechanical and dosimetric characteristics [9]. Their suitability for proton beam calibration is largely influenced by their linear energy transfer (LET) behavior and the shape of the Bragg curves they exhibit under proton irradiation [10,11]. Understanding LET values is crucial, as it directly relates to the biological effectiveness of the radiation dose [12,13]. Similarly, analyzing the Bragg peak positions and widths within these materials allows for precise estimation of beam penetration depth and energy deposition [14]. Consequently, materials that offer predictable and reproducible LET profiles enable more reliable phantom designs and, ultimately, more accurate proton therapy treatments [15].

Recent advancements in machine learning and artificial intelligence have opened new possibilities for modeling complex physical interactions in radiotherapy [16]. To predict physical and chemical properties of polymers, a series of machine learning and deep learning algorithms, including Random Forest (RF), Support Vector Machines, and Linear Regression, have been employed [17]. The algorithms are trained on experimental data derived from known polymers and used to estimate values in less-characterized materials [18]. This approach not only reduces the dependency on labor-intensive experiments but also enhances predictive accuracy by leveraging patterns learned across multiple material datasets [19,20].

The significance of this work lies in its integration of material science, radiation physics, and artificial intelligence to address a critical need in proton therapy: the development of accurate, data-driven models for calibration phantoms. By enabling LET and Bragg curve estimation through trained artificial intelligence (AI) models, the study contributes to more efficient phantom design processes, facilitates rapid evaluation of new polymer candidates, and supports the refinement of treatment planning systems. Ultimately, this research provides a foundational methodology for the broader application of AI in radiological modeling and medical material selection, aligning with ongoing efforts to personalize and optimize cancer therapies. To the best of our knowledge, this is the first study to comprehensively compare a broad range of AI algorithms using LET data derived from proton beam simulations across multiple polymer types while reserving an entirely independent material (Epoxy) for testing generalization performance. Additionally, this study represents the initial utilization of the Locally Weighted Random Forest (LWRF) algorithm for the prediction of Bragg peak positions in polymeric materials based on LET and energy (MeV) profiles. The main objectives of this research are as follows: (i) to train and compare multiple AI models for Bragg peak prediction using high-dimensional LET profiles; (ii) to evaluate model performance on an unseen polymer material to test generalization; (iii) to assess predictive accuracy using both error-based and correlation-based regression metrics; and (iv) to perform statistical significance testing through paired *t*-tests to compare model outputs.

The remainder of this study is organized as follows. Section 2 describes the dataset, the overall framework of the proposed approach, the machine learning and deep learning algorithms employed, and the experimental setup. Section 3 explains the evaluation metrics used to assess the performance of the developed models and to measure the statistical significance of their pairwise differences, followed by a detailed presentation of the empirical results. Finally, Section 4 presents the discussion, future research directions, and conclusions, thereby completing the study.

## 2. Materials and Methods

### 2.1. Data and General Framework

The dataset utilized in this study comprises a total of 120 samples, equally distributed among four polymer groups: Parylene (Specialty Coating Systems, Indianapolis, IN, USA), Lexan (SABIC Innovative Plastics, Pittsfield, MA, USA), Mylar (DuPont Teijin Films, Hopewell, VA, USA), and Epoxy (Sigma-Aldrich, St. Louis, MO, USA), with 30 samples per group. During the model training phase, samples from Parylene, Lexan, and Mylar were employed, whereas Epoxy samples were reserved exclusively for testing purposes to evaluate the generalization capability of the trained models. Each sample is characterized by 201 input features, where the first feature represents the MeV value, and the remaining 200 features correspond to various LET values. The output variable to be predicted is the Bragg peak, which reflects the penetration depth (in mm) of the subatomic particle (proton) within the material. The dataset is publicly available and can be accessed in [21]. The dataset is provided in two separate files as training and testing sets. Each row in the dataset corresponds to one sample. Specifically, each row contains 202 numerical values: the first value represents the energy level (in MeV), the following 200 values represent the corresponding LET distribution, and the final value indicates the Bragg peak position (in mm), which serves as the prediction target in this study.

**Figure 1:**
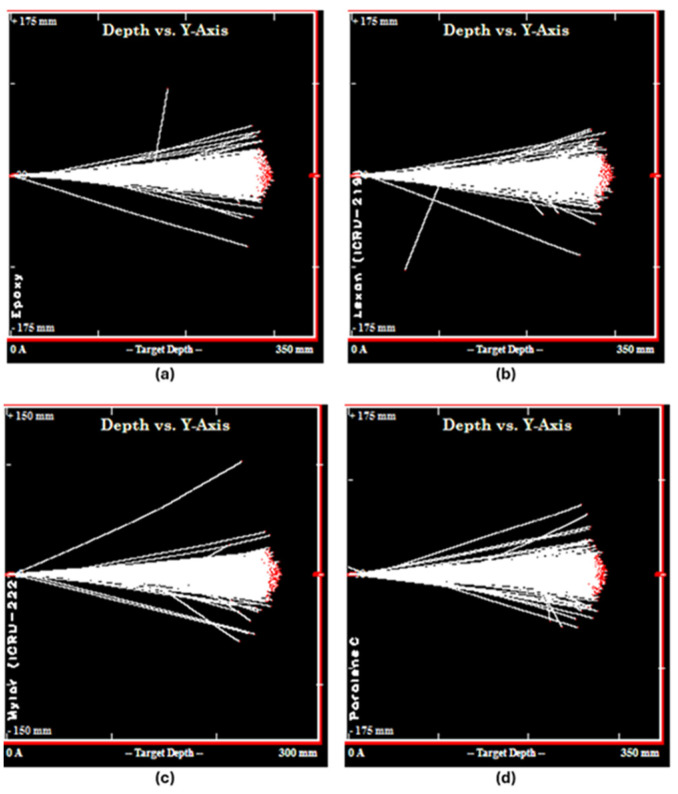
Range and LET views of Epoxy (**a**), Lexan (**b**), Mylar (**c**), and Parylene (**d**) biomaterials used with proton therapy at 230 MeV.

In this study, LET and proton range data of polymeric materials subjected to proton beam irradiation were systematically analyzed to assess dose distribution profiles and penetration depths, as illustrated in Figure 1. Figure 1 depicts the spatial distribution of proton penetration and LET values for Epoxy, Parylene, Lexan, and Mylar polymeric materials at 230 MeV, offering a visual representation of depth–dose relationships critical for Bragg peak prediction modeling. These are not experimental measurements but simulation outputs, which were obtained via Transport of Ions in Matter (TRIM 2013) simulations (SRIM Co., Chester, MD, USA) [11].

The selection of Parylene, Lexan, Mylar, and Epoxy was guided by their established roles as tissue-equivalent materials commonly used in radiological phantom construction. Each of these polymers exhibits distinct LET profiles and mechanical properties, allowing for a diverse assessment of dose deposition behavior under proton irradiation. This diversity is critical for evaluating model generalization across different material types. To ensure controlled, reproducible conditions, TRIM-based simulation outputs were used instead of experimental measurements. This approach also facilitates systematic analysis across a range of energies and material responses.

**Figure 2:**
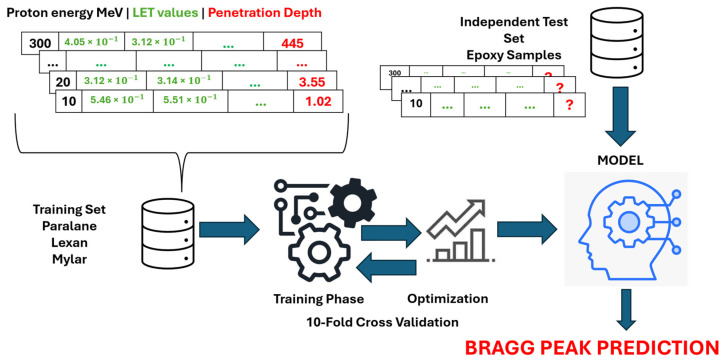
Overview of the framework for Bragg peak value prediction in proton therapy.

The overall framework of the study is illustrated in Figure 2 and can be summarized as follows: Samples in the training set are utilized as inputs for nine algorithms: k-Nearest Neighbors (kNN), Multi-Layer Perceptron (MLP), Support Vector Regression (SVR), RF, Locally Weighted Random Forest (LWRF), Extreme Gradient Boosting (XGBoost), One-dimensional Convolutional Neural Network (1D-CNN), Long Short-Term Memory (LSTM), and Bidirectional Long Short-Term Memory (BiLSTM) to construct predictive models. The hyperparameters of each algorithm are optimized to ensure optimal model performance. The models are then evaluated using an independent test set, where each model predicts the Bragg peak value for the test samples. All analyses were performed using Python version 3.10.13 (Python Software Foundation, Wilmington, DE, USA).

### 2.2. Methods

In order to systematically assess the predictive capabilities of different learning paradigms, both classical machine learning and deep learning models were considered. While canonical machine learning (ML) models are well-suited to structured tabular data, deep learning (DL) models may better capture sequential or spatial dependencies embedded in the LET profiles. This comparison enables a comprehensive understanding of model suitability under identical evaluation criteria. The specific ML and DL algorithms utilized are detailed in the subsequent subsections.

The selection of the machine learning (ML) and deep learning (DL) algorithms in this study was made based on the structural properties of the dataset, the dimensionality of the features, the limited number of samples, and the regression nature of the task. Specifically, each data instance comprises one energy (MeV) value followed by 200 LET values, resulting in a one-dimensional, numerically dense feature vector that contains sequential patterns. To address this, the study included a diverse set of models to evaluate both statistical and temporal learning capabilities.

Canonical ML algorithms such as kNN, SVR, RF, and XGBoost were included due to their proven effectiveness on tabular data with high-dimensional numerical features. These models are known for their generalizability, ease of interpretability, and suitability for relatively small datasets. For instance, SVR offers robust regression capabilities in high-dimensional spaces, while RF and XGBoost are ensemble methods that can handle nonlinear dependencies and variable importance.

Additionally, DL models such as 1D-CNN, LSTM, and BiLSTM were employed to explore the benefits of learning hierarchical and sequential representations from the LET profiles. These models are particularly well-suited for uncovering spatial or temporal dependencies across ordered inputs, which are expected in the distribution of LET values along the proton path. LSTM and BiLSTM were specifically chosen to evaluate the role of memory and bidirectional context in prediction, whereas 1D-CNN was tested for its ability to capture localized activation patterns.

Furthermore, simpler models like MLP were included as baseline neural architectures to examine the effect of depth and connectivity in fully connected networks. By incorporating a spectrum of models with varying levels of complexity and inductive biases, the study provides a comprehensive comparative analysis of model performance on Bragg peak prediction, underpinned by sound methodological justification.

#### 2.2.1. Canonical ML Algorithms

The k-Nearest Neighbors (kNN) algorithm is a simple yet powerful non-parametric method used for both classification and regression tasks. It operates based on the principle that data points with similar features tend to exist in close proximity within the feature space. In the prediction phase, kNN calculates the distance, typically using Euclidean or Manhattan metrics, between the query point and all points in the training set and assigns the output based on the majority (for classification) or average (for regression) of the *k*-nearest neighbors. The algorithm does not require an explicit training phase, which makes it computationally efficient for small to medium-sized datasets. Despite its simplicity, kNN has proven effective in various domains of materials science and polymer engineering due to its ability to capture local structures in data distributions [22,23]. In this study, kNN served as a baseline model to evaluate how a simple, instance-based learner performs on high-dimensional LET data. Its distance-based nature enables it to operate directly on high-dimensional input vectors (i.e., 200 LET features), making it suitable for structured numerical data. Given the sequential structure of the input vectors, kNN relies heavily on the distribution and scale of individual features, which may limit its ability to capture deeper nonlinear trends.

The Multilayer Perceptron (MLP) is a class of feedforward artificial neural networks that has been extensively used for solving both classification and regression problems. It consists of an input layer, one or more hidden layers, and an output layer, with each layer composed of interconnected neurons. The MLP learns complex, non-linear relationships between input and output variables through a supervised learning process known as backpropagation, which minimizes the error by adjusting the network’s weights iteratively. The presence of one or more hidden layers allows MLPs to approximate virtually any continuous function, making them a powerful tool for modeling high-dimensional and non-linear datasets. Activation functions such as ReLU, sigmoid, or tanh are typically employed in hidden layers to introduce non-linearity. Due to its flexibility and effectiveness, MLP has been widely applied across various scientific domains, including materials science and polymer engineering, where capturing intricate patterns in experimental data is essential [24,25,26].

Support Vector Regression (SVR) is a supervised learning algorithm derived from the principles of Support Vector Machines (SVM), designed to perform regression tasks by mapping input features into a high-dimensional space. Unlike traditional regression methods that aim to minimize the error between predicted and actual values, SVR seeks to find a function that deviates from the true values by no more than a predefined margin, ε, while maintaining model simplicity through regularization. This is achieved by minimizing a convex loss function and controlling model complexity via kernel functions such as linear, polynomial, or radial basis function (RBF), which enable the handling of both linear and nonlinear relationships. SVR has demonstrated high generalization capability, making it particularly suitable for applications involving limited or noisy data. Its flexibility and robustness have led to widespread adoption in materials science, engineering, and related fields for property prediction, modeling complex physical systems, and capturing subtle patterns in experimental datasets [27,28,29]. In the context of this study, the structured and moderately high-dimensional input vectors make SVR a suitable candidate due to its proven ability to model nonlinear regression tasks with relatively small datasets and to maintain robustness against overfitting through kernel-based regularization.

Random Forest (RF) is an ensemble learning algorithm widely used for both classification and regression tasks due to its robustness, scalability, and capacity to handle high-dimensional data. It operates by constructing a multitude of decision trees during training and producing either the mode (in classification) or the mean prediction (in regression) of the individual trees as the final output. Each tree is trained on a random subset of the data, and at each node, a random subset of features is considered for splitting, which promotes diversity among trees and helps reduce overfitting. RF does not require extensive parameter tuning and can efficiently manage datasets with missing values or nonlinear relationships. Owing to its strong generalization ability and interpretability via feature importance metrics, Random Forest has been extensively applied in materials science and engineering domains, particularly for property prediction, process optimization, and experimental data modeling [30,31,32]. In this study, RF was selected due to its suitability for high-dimensional, continuous-valued regression problems involving complex feature interactions, as is the case with LET-based Bragg peak prediction. Its ability to generalize well with relatively small datasets and to handle nonlinearity without requiring intensive tuning made it an ideal choice for this task.

Locally Weighted Random Forest (LWRF) is an advanced ensemble learning method that integrates the strengths of RF with local learning principles to improve predictive accuracy, particularly in heterogeneous datasets. Unlike conventional RF models that learn global patterns from the entire training set, LWRF emphasizes local information by assigning weights to training samples based on their proximity to the query point, often measured through distance metrics such as Euclidean distance. During prediction, the algorithm constructs decision trees using weighted subsets of the training data, giving more importance to samples that are closer to the input instance. This localized weighting mechanism enhances the model’s ability to adapt to complex, nonlinear, and region-specific variations in the data. As a result, LWRF offers improved performance in scenarios where data distribution is uneven or when capturing localized phenomena is critical, making it particularly valuable in applications such as materials property prediction, biomedical analysis, and process modeling in manufacturing [33,34]. In the context of this study, LWRF was selected due to the localized and non-uniform nature of the LET distribution across different proton energies and polymer materials. The Bragg peak position is highly sensitive to specific local energy deposition patterns within the LET profile, which may not be captured effectively by global models. By assigning greater weight to training samples that are more similar to the input query—based on distance in high-dimensional feature space—LWRF adapts its regression behavior to locally relevant data regions. This characteristic is particularly valuable when working with moderate-sized datasets and structured sequential features, such as the 200 LET values used in this work. Unlike standard Random Forest, which applies a uniform learning strategy across the entire feature space, LWRF emphasizes local trends and improves prediction accuracy in regions where data distribution is heterogeneous. These properties make LWRF a highly suitable candidate for modeling Bragg peak positions in polymeric materials exposed to proton beams.

Extreme Gradient Boosting (XGBoost) is a highly efficient and scalable ensemble learning algorithm based on gradient boosting decision trees. It is designed to optimize both computational performance and model accuracy, making it well-suited for large-scale and high-dimensional data analysis. XGBoost builds models in a sequential manner, where each new tree is trained to correct the residuals of the previous ensemble, thereby improving predictive performance iteratively. The algorithm includes regularization terms to prevent overfitting and supports parallel processing, sparse data handling, and customizable loss functions. Additionally, it incorporates advanced features such as tree pruning, shrinkage, and column subsampling, which further enhance its robustness and generalization capability. Due to its flexibility and high predictive accuracy, XGBoost has been widely adopted in various scientific and engineering domains, including materials science, for tasks such as property prediction, defect detection, and process optimization [35,36]. In this study, XGBoost was selected due to its ability to model high-dimensional continuous input data with strong regularization mechanisms that are well-suited to preventing overfitting in moderately sized datasets. Its iterative boosting framework makes it particularly effective in capturing complex, nonlinear patterns in LET distributions.

#### 2.2.2. DL Algorithms

One-dimensional Convolutional Neural Networks (1D-CNNs) are a specialized variant of deep learning architectures designed to process and analyze sequential or temporal data, such as time series, signals, or spectra. Unlike traditional feedforward networks, 1D-CNNs utilize convolutional layers that apply learnable filters along one dimension, enabling automatic feature extraction by capturing local patterns and dependencies in the input sequence. These architectures are particularly advantageous in applications where spatial hierarchies or localized features are critical for accurate modeling. The incorporation of pooling layers and non-linear activation functions further enhances the network’s ability to generalize and reduce computational complexity. Due to their efficiency and strong representational power, 1D-CNNs have been widely adopted in various scientific and engineering domains, including materials science, biomedical signal analysis, and structural health monitoring, where they have demonstrated superior performance in tasks such as pattern recognition, anomaly detection, and property prediction [37,38,39]. In this study, the LET values represent an ordered sequence of energy deposition across tissue depth, making 1D-CNN architectures suitable for capturing local patterns and transitions along the LET profile. The convolutional filters in 1D-CNN are able to identify feature motifs that correspond to specific spatial structures related to the Bragg peak position.

Long Short-Term Memory (LSTM) networks are a specialized type of recurrent neural network (RNN) designed to learn and retain long-term dependencies in sequential data. Unlike traditional RNNs, which suffer from vanishing or exploding gradient issues during training, LSTM networks incorporate gated mechanisms—namely the input gate, forget gate, and output gate—that regulate the flow of information and enable the network to maintain memory over extended time intervals. This architecture makes LSTM particularly well-suited for modeling complex temporal patterns in time series, signal processing, and sequential decision-making tasks. In scientific and engineering domains, LSTM has demonstrated strong performance in applications such as fault diagnosis, predictive maintenance, and material property forecasting, where temporal dependencies are crucial. The ability of LSTM networks to model nonlinear relationships and capture dynamic system behavior makes them a powerful tool for handling high-dimensional and temporally rich datasets encountered in modern research [40,41]. In this study, the LET profile is treated as a sequential feature vector of length 200, where each element represents energy deposition at increasing depth. This ordered structure allows LSTM to model dependencies across depth-wise energy distribution, even though the sequence is spatial rather than temporal in origin.

Bidirectional Long Short-Term Memory (BiLSTM) networks are an advanced extension of standard LSTM architectures, designed to capture both past and future temporal dependencies within sequential data. Unlike conventional LSTM, which processes information in a single forward direction, BiLSTM consists of two parallel LSTM layers that operate in opposite directions—one from past to future and the other from future to past—allowing the model to utilize context from both preceding and succeeding time steps. This bidirectional processing enhances the model’s ability to learn richer and more informative representations, particularly in tasks where the full sequence context is important. BiLSTM networks have been successfully applied in domains such as natural language processing, biomedical signal analysis, and time-series prediction. In materials science and polymer engineering, BiLSTM has demonstrated strong potential for modeling complex temporal phenomena such as dynamic mechanical behavior, degradation processes, and real-time sensor monitoring. The enhanced contextual awareness offered by BiLSTM makes it an effective tool for predictive modeling in systems characterized by sequential dependencies and temporal complexity [41,42,43]. In this study, BiLSTM was employed to explore the potential benefits of modeling the LET profile in both forward and reverse directions. Since the Bragg peak may be influenced by patterns that span across the entire LET sequence—not just in one directional flow—bidirectional modeling allows the network to leverage complete contextual information when mapping energy deposition profiles to penetration depth.

### 2.3. Experimental Setup

The dataset was partitioned into 75% for training and 25% for independent test purposes. Specifically, the training set includes 90 samples (30 from each of Parylene, Lexan, and Mylar), while the test set includes 30 entirely unseen samples from Epoxy. No Epoxy samples were used during the training or validation phases. This separation was intended to evaluate the generalization performance on a polymer type not seen during training. A 10-fold cross-validation approach was applied on the training set to optimize the hyperparameters of several canonical machine learning and deep learning algorithms. The 10-fold cross-validation method is a widely used resampling technique for model evaluation and hyperparameter tuning in machine learning. In this method, the training dataset is partitioned into ten equal-sized folds. During each iteration, one fold is reserved for validation while the remaining nine folds are used for training. This process is repeated ten times, with each fold used exactly once for validation. In addition, the samples within the folds are randomly selected in each epoch. To maintain balanced representation, each fold in the cross-validation process includes an equal number of samples from the three polymer types: Parylene, Lexan, and Mylar. The final performance metric is obtained by averaging the results across all folds, which helps to reduce variance and provides a more robust estimate of the model’s generalization ability. This approach is particularly effective for mitigating overfitting and ensuring that the model performs well on unseen data [44].

To prevent any risk of data leakage and to evaluate the generalization capability of the models, the Epoxy samples were strictly excluded from the training and validation phases. Specifically, only the Parylene, Lexan, and Mylar samples were used in the 10-fold cross-validation and hyperparameter tuning processes. The Epoxy dataset was entirely reserved for independent testing, ensuring that the models were not exposed to any Epoxy-related information during training. This separation enables a robust assessment of how well the models can generalize to an unseen polymer material and confirms the integrity of the evaluation protocol.

In this study, hyperparameter optimization for all machine learning and deep learning models was conducted using a grid search strategy. For each candidate configuration in the search space, a 10-fold cross-validation technique was applied on the training dataset to ensure robust model evaluation and to mitigate overfitting. During this process, the correlation coefficient (*CC*) was used as the primary performance metric, and the optimal hyperparameter set for each algorithm was determined by selecting the configuration that yielded the highest average *CC* across the validation folds. This systematic integration of grid search with cross-validation enabled a fair and reproducible comparison among the candidate models.

In the training stage, hyperparameter optimization was performed with the objective of maximizing the correlation coefficient (*CC*) metric. For kNN, the number of nearest neighbors, k, was set to 1 [range: 1–89], and Euclidean distance was determined from several distance metrics, including Euclidean, Manhattan, Chebyshev, and Minkowski distances. The MLP algorithm was configured with one hidden layer comprising 5 neurons [range: 1–150] [45]. The model incorporated the sigmoid function as its activation function, and a dropout rate of 0.3 was utilized. For the SVR algorithm, the optimal *CC* value was achieved using ε-SVR with a linear kernel (linear, polynomial, radial basis, and sigmoid kernels were utilized for both ε-SVR and ν-SVR in the grid search), where the epsilon parameter in the loss function was set to 0.1 [ε ∈ {0.0001, 0.001, 0.01, 0.05, 0.1, 0.2, 0.5}] [46]. For the RF algorithm, the optimal number of trees was identified as 400 within the tested range of 100 to 1000 [47]. For the LWRF algorithm, the optimal number of trees was determined as 100, and the number of neighbors used for local weighting was set to 55 [47]. For the XGBoost algorithm, the hyperparameters maximum depth [range: 3–10], gamma [range: 0–0.5], alpha [∈ {0, 0.001, 0.005, 0.01, 0.05, 0.1, 0.5, 1, 5, 10}], and number of estimators [range: 10–1000] were tuned to optimal values of 3, 0.1, 0, and 30, respectively [48].

The 1-D CNN model was optimized with 10 layers [range: 1–20], 64 filters [range: 1–128], and a kernel size of 3. In the LSTM model, the number of layers was optimized to 15 [range: 1–20], and the number of units (memory cells) was set to 128 [range: 1–256]. On the other hand, the BiLSTM model was optimized with 15 layers and 64 units (memory cells).

The 1D-CNN model was designed incorporating batch normalization and spatial dropout 1D for regularization. Notably, spatial dropout 1D operates by randomly deactivating entire feature map channels of a convolutional layer, rather than individual neurons. Spatial dropout 1D was applied with a rate of 0.3 [optimized in range: 0.1–0.5]. Within the LSTM and Bi-LSTM architectures, the recurrent dropout strategy was adopted. This particular regularization technique involves applying an identical dropout mask consistently throughout the recurrent connections, which is crucial for mitigating information loss typically associated with standard dropout in recurrent neural networks. It effectively curbs overfitting and is widely recognized as the most suitable dropout variant for RNNs. A recurrent dropout rate of 0.3 was determined for this work. Additionally, the ReLU activation function was employed for all deep learning architectures.

All deep learning models, including LSTM, BiLSTM, and 1D-CNN, were implemented using a uniform training framework. Each network was trained with a batch size of 8 and a maximum epoch count of 50. An early stopping callback was incorporated: if the validation metric failed to improve for five consecutive epochs, training was halted automatically. The optimizer employed in all cases was Adam, configured with a learning rate of 0.001. Model optimization targeted the mean squared error (MSE) loss throughout. Alternative optimizers and loss functions were not explored in this study due to computational restrictions.

To provide a transparent comparison of computational costs, the total number of trainable parameters was calculated for each deep learning model architecture utilized in this study:

The 1D-CNN comprised 10 sequential convolutional layers, each with 64 filters of size 3, followed by a fully connected regression output layer. Given an input length of 201, the total number of trainable parameters was 124,289. The primary contributors to this count were the large number of filters and the fully connected output, both of which increase model expressiveness but also computational burden. The LSTM model consisted of 15 stacked recurrent layers, each containing 128 memory cells. The total number of trainable parameters was computed as 2,011,265. This high parameter count results from the recurrent gating mechanisms (four gates per cell) and the depth of the architecture, leading to substantial memory and computation requirements during both training and inference. The bidirectional LSTM (BiLSTM) architecture mirrored the unidirectional LSTM in layer count (15) but used 64 memory cells in each direction per layer. The bidirectional configuration effectively doubles the number of trainable parameters per layer. The total parameter count for the BiLSTM model was 1,061,993. This results in significantly higher computational and memory demands compared to standard LSTM, particularly during training, as both forward and backward passes are required for every sequence. Overall, the LSTM and BiLSTM models entailed markedly higher computational complexity than the 1D-CNN, both in terms of parameter space and practical training requirements (e.g., RAM/VRAM and CPU/GPU utilization). Such complexity may be prohibitive in environments without GPU acceleration or with limited training data, increasing the risk of overfitting.

In this study, the LET values and MeV are treated as a one-dimensional structured sequence for each sample. Specifically, each input instance consists of 200 LET values (treated as a sequence) following one MeV value, resulting in an ordered input of 201 features. This design allows sequence-based models such as 1D-CNN, LSTM, and BiLSTM to learn temporal/spatial dependencies across the LET profile.

Prior to training the 1D-CNN model, all feature vectors were normalized to the range of 0 to 255. This normalization was motivated by the fact that convolutional neural networks were originally designed for image processing tasks, where input pixel intensities typically span the 0–255 range in 8-bit grayscale images. By aligning the scale of input features with the expected distribution of image-like data, the convolutional filters in the CNN are able to better learn local patterns and hierarchical representations. Moreover, this preprocessing step ensures numerical stability during training and facilitates more effective gradient propagation across convolutional layers.

For traditional machine learning models (e.g., SVR, RF, LWRF, XGBoost, and kNN), no normalization was applied to the input features. This decision was based on the nature of these models: ensemble-based methods such as Random Forest and XGBoost are inherently insensitive to feature scale, and kNN, LWRF, and SVR were implemented using Euclidean distance with consistent dimensionality and range across features, eliminating the need for additional scaling in this specific dataset. Normalization was not applied to the LSTM and BiLSTM inputs, as the LET vectors are numerically stable, uniformly structured across samples, and physically meaningful in their original scale. Preserving the true magnitude and progression of energy deposition was prioritized to retain the spatial relationships required for accurate sequential modeling.

No feature selection was performed, as each LET channel corresponds to a physically meaningful spatial position in the material. Removing any of these values could potentially disrupt the sequential structure required for both deep and non-deep models to accurately predict the Bragg peak location.

The training set comprises 90 samples, equally distributed across three polymer types (Mylar, Lexan, and Parylene), while the test set includes 30 independent Epoxy samples. As the task involves regression with continuously distributed target values and no observable imbalance, stratification was not applied during 10-fold cross-validation.

The use of the correlation coefficient (*CC*) as the common performance metric for hyperparameter tuning offers several advantages in the context of this study. First, *CC* directly quantifies the linear relationship between the predicted and actual values, making it a robust indicator of the model’s ability to preserve relative ordering and distribution trends—an especially valuable trait in regression problems involving physical phenomena such as proton range or energy deposition. Second, *CC* is scale-invariant, meaning it remains unaffected by differences in magnitude or unit scaling across datasets, which is particularly advantageous when comparing models trained on normalized versus non-normalized features. Third, *CC* provides a bounded and interpretable range [−1, 1], facilitating consistent evaluation across diverse model architectures. Although other error-based metrics such as *MAE* or *RMSE* offer insights into absolute prediction deviations, the use of *CC* ensures that model optimization remains focused on maximizing predictive consistency and general linear agreement, which was the primary goal of this investigation.

### 2.4. Evaluation Metrics

Correlation Coefficient (*CC*), Coefficient of Determination (*R*^2^), Mean Absolute Error (*MAE*), Root Mean Squared Error (*RMSE*), Relative Absolute Error (*RAE*), and Root Relative Squared Error (*RRSE*) are widely used to evaluate the accuracy of predictions in regression tasks. The subsequent paragraphs will elucidate the definitions and corresponding formulas for the metrics in question.

*CC* measures the linear relationship between predicted and true values. It is also known as Pearson’s correlation coefficient. The value of the correlation coefficient lies between −1 and 1. A higher magnitude of *CC* (closer to 1 or −1) indicates a stronger linear relationship between the predicted and actual values. A value of 1 represents a perfect positive linear correlation between predictions and true values, −1 represents a perfect negative linear correlation, and 0 represents no linear correlation between predictions and true values. The formula for *CC* is given in Equation (1):

(1) $$CC\;=\;\frac{\sum_{i=1}^{N}\left(y_{i}-\bar{y})({\hat{y}}_{i}-\bar{\hat{y}}\right)}{\sqrt{\sum_{i=1}^{N}{\left(y_{i}-\bar{y}\right)}^{2}}\sqrt{\sum_{i=1}^{N}{\left({\hat{y}}_{i}-\bar{\hat{y}}\right)}^{2}}}$$

where $y_{i}$, ${\hat{y}}_{i}$, $\bar{y}$, $\bar{\hat{y}}$, and N represent the actual value, predicted value, mean of actual values, mean of predicted values, and number of observations, respectively.

$R^{2}$ is a statistical measure that evaluates the proportion of variance in the dependent (target) variable that is explained by the regression model, regardless of the number of predictors. It provides an indication of the model’s goodness of fit. *R*^2^ can be formally defined as the square of the *CC* between the actual values $y_{i}$ and the predicted values ${\hat{y}}_{i}$, as follows:

(2) $$R^{2}=\;{\left(Corr\left(y,\hat{y}\right)\right)}^{2}\;=\;{\left(\frac{\sum_{i=1}^{N}\left(y_{i}-\bar{y})({\hat{y}}_{i}-\bar{\hat{y}}\right)}{\sqrt{\sum_{i=1}^{N}{\left(y_{i}-\bar{y}\right)}^{2}}\sqrt{\sum_{i=1}^{N}{\left({\hat{y}}_{i}-\bar{\hat{y}}\right)}^{2}}}\right)}^{2}$$

$R^{2}$ values range from 0 to 1, where a value closer to 1 indicates that a greater proportion of variance is explained by the model. While the *CC* quantifies the linear association between predicted and actual values, $R^{2}$ is more commonly used in regression tasks as it indicates the proportion of variance in the target variable explained by the model. Moreover, $R^{2}$ is always non-negative and directly reflects model performance, making it more interpretable in predictive contexts.

The *MAE* represents the average magnitude of errors in a set of predictions, without considering their direction. A value of 0 indicates perfect predictions, with higher values showing larger errors. It does not provide information about the direction of the errors, only their magnitude. The formula for *MAE* is given in Equation (3):

(3) $$MAE\;=\;\frac{1}{N}\sum_{i=1}^{N}\left|y_{i}-{\hat{y}}_{i}\right|$$

where $y_{i}$, ${\hat{y}}_{i}$, and N represent the actual value, predicted value, and number of observations, respectively.

The *RMSE* measures the square root of the average squared differences between predicted and actual values, emphasizing larger errors. A value of 0 indicates perfect predictions. Higher values suggest more significant prediction errors, with an emphasis on larger deviations. The formula for *RMSE* is given in Equation (4):

(4) $$RMSE\;=\;\sqrt{\frac{1}{N}}\sum_{i=1}^{N}{\left(y_{i}-{\hat{y}}_{i}\right)}^{2}$$

where $y_{i}$, ${\hat{y}}_{i}$, and N represent the actual value, predicted value, and number of observations, respectively.

The *RAE* normalizes the total absolute error by the total absolute deviation from the mean of the actual values. A value of 0 represents perfect predictions. When the value of *RAE* is equal to 1, the model’s predictions are as accurate as simply predicting the mean of the actual values. A value less than 1 signifies that the model’s predictions are better than predicting the mean, whereas a value greater than 1 signifies that the model’s predictions are worse than predicting the mean. The formula for *RAE* is given in Equation (5):

(5) $$RAE\;=\;\frac{\sum_{i=1}^{N}\left|y_{i}-{\hat{y}}_{i}\right|}{\sum_{i=1}^{N}\left|y_{i}-\bar{y}\right|}$$

where $y_{i}$, ${\hat{y}}_{i}$, $\bar{y}$, and N represent the actual value, predicted value, mean of actual values, and number of observations, respectively.

The *RRSE* normalizes the *RMSE* by the root of the mean squared deviation from the mean of the actual values. Similarly to the *RAE* metric, a value of 0 represents perfect predictions. The model performs no better than predicting the mean when *RRSE* has a value of 1. For values greater than 1, the model performs worse than predicting the mean. The formula for *RRSE* is given in Equation (6):

(6) $$RRSE\;=\;\sqrt{\frac{\sum_{i=1}^{N}{\left(y_{i}-{\hat{y}}_{i}\right)}^{2}}{\sum_{i=1}^{N}{\left(y_{i}-\bar{y}\right)}^{2}}}$$

where $y_{i}$, ${\hat{y}}_{i}$, $\bar{y}$, and N represent actual value, predicted value, mean of actual values, and number of observations, respectively.

The paired *t*-test checks whether the actual and predicted values differ significantly. In a paired *t*-test, t-statistic and *p*-value evaluation metrics are utilized to interpret statistical significance. The t-statistic indicates how many standard deviations the difference between the actual and predicted values is from zero. The *p*-value tells us whether the difference is statistically significant. The larger the absolute value of the t-statistic, the stronger the evidence that there is a significant difference between the actual and predicted values. If the *p*-value is less than 0.05, it can be concluded that the differences between the actual and predicted values are statistically significant. The t-statistic helps quantify the strength of the evidence against the null hypothesis, which usually states that there is no significant difference between the actual and predicted values. The t-statistic formula is given in Equation (7):

(7) T = d¯sdn

where $\bar{d}$, $s_{d}$, and n represent the mean of the differences between the paired samples, the standard deviation of the differences between the paired samples, and the number of paired observations, respectively. The *p*-value is the probability of observing a test statistic as extreme as (or more extreme than) the calculated t-value, assuming the null hypothesis is true. It is computed using the t-distribution. The *p*-value is the cumulative probability of the t-statistic from the t-distribution table and given in Equation (8):

(8) $$p-value\;=\;P\left(T\geq t\right)$$

where T and t represent the theoretical random variable following the t-distribution and the calculated t-statistic based on the sample data used to test the hypothesis, respectively.

A paired *t*-test is employed to determine whether there is a statistically significant difference between the regression models. Given a set of paired predictions, the t-statistic is calculated as shown in Equation (7). In this context, the key distinction lies in computing the differences between the predictions produced by the two regression models (9).

(9) $$d_{i}=p_{i}^{\left(1\right)}-p_{i}^{\left(2\right)}$$

where $p_{i}^{\left(1\right)}$, $p_{i}^{\left(2\right)}$, and $d_{i}$ represent the prediction of regression model 1 on instance i, the prediction of regression model 2 on the same instance, and the difference in predictions for each test sample, respectively.

## 3. Results

Table 1 presents a comparative evaluation of nine machine learning and deep learning models based on six standard regression performance metrics: *CC*, *MAE*, *RMSE*, *RAE*, *RRSE*, and *R*^2^.

**Figure 3:**
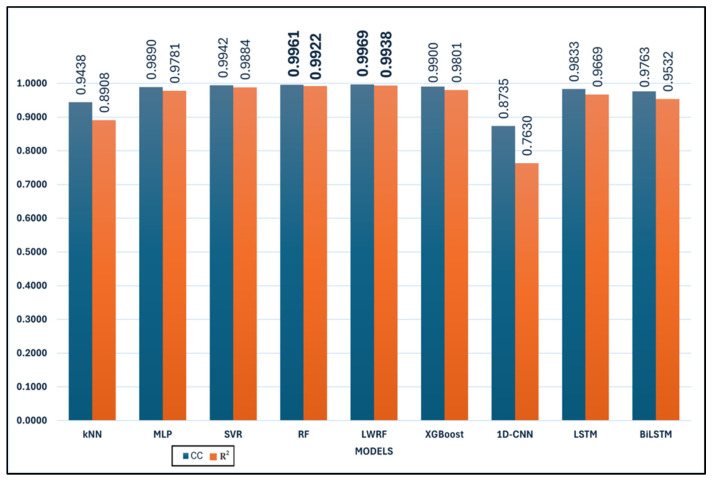
Comparative analysis of machine learning and deep learning models for Bragg peak prediction based on *CC* and *R^2^* evaluation metrics (**bold** values represent the highest performance).

**Figure 4:**
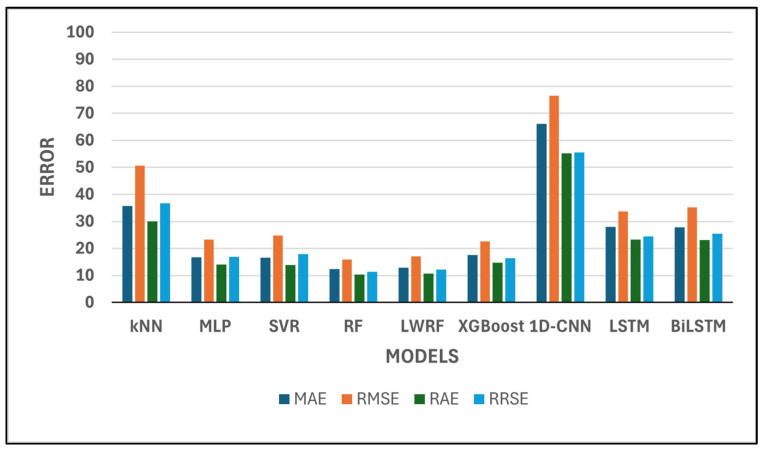
Comparative analysis of error metrics—*MAE*, *RMSE*, *RAE*, and *RRSE*—across machine learning and deep learning models for Bragg peak prediction.

Among all models, RF and LWRF consistently demonstrate the most favorable performance across all metrics. In particular, RF achieves the lowest *MAE* (12.32), *RMSE* (15.82), *RAE* (10.35), and *RRSE* (11.44), while also attaining a high *CC* of 0.9961 and an *R*^2^ value of 0.9922 (Figure 3 and Figure 4). However, LWRF outperforms RF with a *CC* of 0.9969 and an even higher *R*^2^ value of 0.9938, indicating a slightly stronger correlation with the ground truth. Although LWRF achieves the highest correlation coefficient (*CC* = 0.9969) and determination coefficient (*R^2^* = 0.9938), the RF model yields lower *MAE* and *RMSE* values. This apparent discrepancy is not contradictory, as correlation-based metrics (*CC* and *R*^2^) reflect the directional consistency and variance explanation between predicted and actual values, whereas error-based metrics (*MAE* and *RMSE*) quantify the magnitude of deviation. A model can therefore exhibit strong correlation with the target while still producing slightly larger absolute errors in certain instances. The difference in *CC* and *R^2^* between LWRF and RF, while numerically present, was not statistically significant (as indicated by *p*-values > 0.05). Thus, these small differences may be attributed to data variability rather than a true performance advantage.

According to Figure 3 and Figure 4, a quantitative comparison of model performance reveals substantial differences across the evaluated algorithms. For example, the mean absolute error (*MAE*) values range from as low as 12.32 for Random Forest (RF) to as high as 66.10 for 1D-CNN, corresponding to more than a fivefold increase in prediction error between the best- and worst-performing models. Similarly, the correlation coefficient (*CC*) spans from 0.8735 (1D-CNN) to 0.9969 (LWRF), highlighting a significant gap in predictive ability. The top two models, RF and LWRF, exhibit closely matched performance, with their *MAE* values differing by less than 0.5 and their *CC* values by only 0.0008, indicating very limited variance between these leading approaches. In contrast, traditional models such as kNN display a much wider deviation, with an *MAE* of 35.7663 and a *CC* of 0.9438, further illustrating the performance variability observed in this study. Overall, the range of evaluation metrics across all models clearly demonstrates both the statistical variance and the comparative strengths and weaknesses among the examined algorithms.

The SVR and MLP models also perform well, with relatively low error metrics and high correlation coefficients. SVR, for instance, achieves a *CC* of 0.9942 and an *R*^2^ of 0.9884, reflecting its reliability in approximating actual values.

While XGBoost achieves a high correlation coefficient (*CC* = 0.9900) and a respectable *R*^2^ score (0.9801), its error metrics (*MAE* = 17.58, *RMSE* = 22.70) are slightly higher than those of MLP and SVR. This suggests that although XGBoost is capable of producing outputs that closely follow the overall trend of the data, its pointwise accuracy is relatively less precise in this task compared to the top performers. In contrast, kNN demonstrates the lowest predictive accuracy among the classical machine learning models considered. With a *CC* of 0.9438 and an *R*^2^ of 0.8908, alongside relatively high error values (*MAE* = 35.77, *RMSE* = 50.70), kNN’s performance indicates limited suitability for this regression problem, possibly due to its local averaging nature, which may struggle with global nonlinearities in the data. However, model performance is known to be highly sensitive to the number of neighbors (k) and the distance metric employed. In this work, k-values ranging from 1 to 89 were evaluated through grid search, with the optimal result obtained at k = 1. It should be noted that, due to the limited size of the training set (90 samples in total), the maximum possible value for k is inherently constrained to 89. This restriction may have limited the algorithm’s ability to fully leverage neighborhood-based information, as larger training sets typically allow for a broader and potentially more informative selection of neighbors. In addition, several distance metrics were assessed, including Euclidean, Manhattan, Chebyshev, and Minkowski distances, to examine their influence on predictive accuracy. These parameter variations did not result in any substantial improvement in kNN performance, and the overall model ranking remained unchanged.

Conversely, 1D-CNN shows the weakest performance among all models. It records the highest error values (*MAE* = 66.10, *RMSE* = 76.59, *RAE* = 55.20, *RRSE* = 55.50) and the lowest correlation metrics (*CC* = 0.8735, *R*^2^ = 0.763), suggesting poor generalization ability in this context. The extremely poor performance of the 1D-CNN model observed in this study warrants further consideration of architectural optimization. The implemented 1D-CNN architecture consisted of 10 convolutional layers, each with 64 filters and a kernel size of 3. The Rectified Linear Unit (ReLU) activation function was applied throughout all convolutional layers to introduce non-linearity. Batch normalization and spatial dropout (rate 0.3) were employed to mitigate overfitting and stabilize the learning process. Despite these measures, the 1D-CNN model failed to achieve satisfactory results, with *MAE* and *RMSE* values (66.10 and 76.59, respectively) far exceeding those of the other evaluated models. Several factors may have contributed to this underperformance. First, the relatively limited size of the dataset (only 90 training samples) likely constrained the model’s ability to generalize, as deep learning architectures such as 1D-CNN generally require substantially larger training sets to effectively capture relevant patterns. Second, additional architectural hyperparameters, such as the number of layers, kernel size, number of filters, stride, and choice of activation function, are known to significantly influence 1D-CNN model capacity and performance in regression tasks. In this study, only the ReLU activation function was implemented, and the impact of alternative activation functions (e.g., Leaky ReLU, ELU, or tanh) could not be assessed due to computational resource limitations. Third, all deep learning experiments in this study were executed on a CPU platform, which imposed a significant computational limitation on the scope of hyperparameter optimization. Consequently, the range of tested hyperparameters (e.g., filter numbers, kernel sizes, layer counts, dropout rates, and activation function types) could not be extensively explored, and exhaustive tuning was not feasible under these resource constraints.

Recurrent models such as LSTM and BiLSTM offer moderate performance, with *R*^2^ values of 0.9669 and 0.9532, respectively. Although their correlation coefficients are high (above 0.97), the magnitude of their errors remains larger than those of tree-based ensemble methods. To ensure a fair and reproducible comparison among the deep learning models, all architectures (LSTM, BiLSTM, and 1D-CNN) were trained under the same protocol. The maximum number of epochs was set to 50. An early stopping mechanism was applied, whereby training was terminated if no improvement in validation performance was detected over five consecutive epochs. A batch size of eight was employed for all models, and the Adam optimizer was utilized with a learning rate of 0.001. The mean squared error (MSE) was used as the loss function. These training parameters were selected to provide a balanced approach between computational efficiency and model convergence and to enable fair performance comparisons among the evaluated deep learning methods. It should be acknowledged, however, that the potential impact of alternative optimizers, such as AdamW or RMSprop, could not be assessed within the scope of this study due to computational resource limitations. Additionally, only the mean squared error (MSE) loss function was employed in model training. The effects of alternative loss functions, such as *MAE*, Huber loss, or log-cosh loss, were not investigated.

Overall, the results indicate that ensemble-based models, particularly RF and LWRF, offer superior predictive accuracy and generalization in this regression task. Deep learning models, especially 1D-CNN, appear to be less suitable in their current configurations, potentially requiring further optimization or more training data.

In order to assess the statistical reliability of each model’s predictions, a paired *t*-test was performed by comparing the actual and predicted values across 30 instances. The difference was defined as actual minus predicted, and the resulting t-statistics and *p*-values are summarized in Table 2. Some of the *p*-values in the table are written in bold, indicating that the difference between the actual and predicted values is statistically significant.

According to the results, several models exhibited a statistically significant tendency to overestimate the actual values, including RF, LWRF, XGBoost, and BiLSTM (p < 0.05). Among them, LWRF showed the strongest overestimation effect with a highly significant *p*-value ($4.23\times 10^{-6}$). In contrast, the SVR model significantly underestimated the actual values (t = 4.0962, *p* = 0.0003). On the other hand, models such as kNN, MLP, 1D-CNN, and LSTM showed no statistically significant difference between their predictions and the actual values (*p* > 0.05). This may indicate that these models do not exhibit a consistent tendency to either overestimate or underestimate the true values. However, it should be noted that a non-significant *p*-value (*p* > 0.05) does not necessarily imply the absence of prediction bias or error. Rather, it indicates that no statistically significant difference could be detected under the present experimental conditions and sample size. Therefore, lack of significance should be interpreted with caution and does not equate to a guarantee of unbiased model performance.

Table 3 summarizes the pairwise statistical comparison of models using the paired *t*-test applied to their prediction differences. In the table, the vertically aligned cells for each comparison represent the t-statistic, *p*-value, and confidence interval, respectively. Bold values (i.e., p < 0.05) indicate that the difference in model predictions is statistically significant. Table 4 presents a comparison of the models based on the *CC* metric. Bold arrows are included to indicate a statistically significant difference between the results of two models. An ↑ (upward arrow) means the model indicated by the arrowhead has a higher *CC*. A ← (leftward arrow) likewise shows that the model pointed to by the arrow has a higher *CC*. In this way, statistical significance (p-value) and performance ranking (based on *CC*) are presented separately.

When kNN and MLP are compared, the positive t-statistic and the 95% confidence interval for the mean difference ([4.1968, 41.6438]) reveal that the average predictions of the kNN model are significantly higher than those of the MLP model. While the paired *t*-test highlights that the kNN model produces significantly higher average predictions, the superior correlation coefficient of the MLP model suggests it is a more suitable choice when the objective is to maximize the relationship with the true values and explain the variance in the dependent variable. Based on the test results comparing kNN and SVR, the paired *t*-test revealed a statistically significant difference in the predictions of the two models (t = 3.4631, *p* = 0.0016793), with the average predictions of kNN being significantly higher than those of SVR (95% CI for the mean difference [13.0973, 50.881]). However, a crucial factor in model selection for maximizing *CC* (or *R*^2^) is the strength of the correlation between the model’s predictions and the actual values. The *CC* for SVR (0.9942) is substantially higher than that of kNN (0.9438). This indicates that the predictions generated by the SVR model exhibit a much stronger linear relationship with the true values. The enhanced correlation suggests that SVR is better at capturing the underlying patterns in the data and explaining the variance in the dependent variable, ultimately leading to a better model fit despite its lower average predictions relative to kNN. In the kNN–RF comparison, the t-statistic of 0.8831 is close to zero, suggesting a minimal difference between the means of the two prediction sets relative to their variability. The 95% confidence interval for the mean difference between the predictions of KNN and RF is [−9.4446, 23.7978]. The inclusion of zero within this interval further supports the conclusion that there is no statistically significant difference in the average predictions of the two models. The RF model exhibits a considerably higher *CC* (0.9961) compared to the kNN model (0.9438). This indicates a much stronger linear relationship between the predictions of the RFF model and the actual values. Given the objective of maximizing the *R*^2^, the RF model can be the preferred choice despite the lack of a statistically significant difference in average predictions between the two models. In the kNN–LWRF comparison, the t-statistic of 0.5762 indicates a minimal difference between the mean predictions of the two models relative to the variability in the data. The *p*-value of 0.5689 is greater than the conventional significance level of 0.05. This suggests that there is no statistically significant difference between the average predictions of the kNN and LWRF models. The observed difference is likely due to random variation. The 95% confidence interval for the mean difference between the predictions of kNN and LWRF is [−11.8694, 21.1806]. The inclusion of zero within this interval further supports the conclusion that there is no statistically significant difference in the average predictions of the two models. While the paired *t*-test indicates no statistically significant difference in the average predictions between kNN and LWRF, the LWRF model exhibits a substantially higher *CC* (0.9961) compared to kNN (0.9438). Despite the lack of a statistically significant difference in average predictions, the LWRF model can be the preferred choice over kNN when the objective is to maximize the *CC* or *R*^2^. The application of a paired *t*-test showed no statistically significant difference in the average predictions generated by the kNN and XGBoost models. The t-statistic of 0.668 indicates that the magnitude of the difference between the average predictions of kNN and XGBoost is small relative to the variability within the prediction sets. A t-statistic near zero suggests that the sample means of the two prediction sets are not substantially different. The *p*-value of 0.5094 signifies that there is no statistically significant difference in the average predictions of the two models. The 95% confidence interval for the mean difference between the predictions of kNN and XGBoost is [−11.3589, 22.3783]. This further supports the conclusion that there is no statistically significant difference in the average predictions of kNN and XGBoost. Nevertheless, the XGBoost model exhibits a higher *CC* (0.99) in comparison to kNN (0.9438). This enhanced correlation signifies a stronger linear association with the actual values for XGBoost, making it the preferred model for maximizing the *CC* and *R*^2^ as it suggests a greater capacity to account for data variance, notwithstanding the lack of a statistically significant difference in average predictions. Based on the paired *t*-test results comparing kNN and 1D-CNN, the t-statistic of −0.1823 is very close to zero, indicating a negligible difference between the mean predictions of the two models relative to the data’s variability. The *p*-value of 0.8566 suggests that there is no statistically significant difference between the average predictions of the kNN and 1D-CNN models. The 95% confidence interval for the mean difference between the predictions of kNN and 1D-CNN is [−35.0216, 29.2883]. The inclusion of zero within this interval strongly supports the conclusion that there is no statistically significant difference in the average predictions of the two models. Although the paired *t*-test indicates no statistically significant difference in average predictions, the kNN model exhibits a higher *CC* (0.9438) compared to the 1D-CNN (0.8735). This implies that kNN demonstrates a stronger linear relationship with the actual values and is likely to explain a greater proportion of the variance in the dependent variable, leading to a higher *R*^2^ despite the statistically similar average predictions. According to the results between kNN and LSTM, the t-statistic of 1.177 and *p*-value of 0.2488 suggest that there is no statistically significant difference between the kNN and LSTM models in terms of their predictions. The confidence interval ranging from −8.16207 to 30.2927, which includes zero, further confirms that the difference between the two models is not statistically significant. Since LSTM has a higher *CC* (0.9833), it suggests that LSTM’s predictions are more consistent with the true values, which is the goal if maximizing the *CC* is aimed for. In the kNN–BiLSTM comparison, the t-statistic (−0.1887) indicates no significant difference between the predictions of kNN and BiLSTM. The *p*-value (0.85162) confirms that the difference in performance between the two models is not statistically significant. Thus, it cannot be concluded that one model outperforms the other. The confidence interval (−19.7046, 16.3752) includes zero, reinforcing the idea that the difference in performance could be negligible. However, since BiLSTM has a higher *CC* (0.9763) compared to kNN (0.9438), BiLSTM can be considered slightly better in terms of prediction accuracy, although the performance difference is not statistically significant.

In the MLP–SVR comparison, a positive t-statistic (3.5596) indicates that the predictions of the MLP model are significantly higher than the predictions of the SVR model. This suggests that, on average, MLP’s predictions are higher compared to SVR’s predictions. The *p*-value (0.001303) is less than 0.05, confirming that the difference in predictions between MLP and SVR is statistically significant and not due to random chance. Therefore, the result suggests that MLP’s predictions are significantly higher than those of SVR. The 95% confidence interval for the mean difference between MLP and SVR ([3.85813, 14.2795]) indicates that the true mean difference in predictions falls within this range and is positive. This confirms that SVR’s predictions are generally lower than MLP’s predictions. While the *t*-test suggests that MLP’s predictions are higher, it does not imply that MLP is the better model. The higher *CC* of SVR indicates that SVR provides more accurate predictions, making it the preferable model in terms of aligning with the true values. Based on the MLP-RF comparison, the negative t-statistic (−4.3858) tells us that RF’s predictions are higher than MLP’s, and the *p*-value (0.00013914) confirms that the difference is statistically significant, meaning it is unlikely to be due to random chance. The 95% confidence interval for the mean difference in predictions between MLP and RF is [−23.0854, −8.40197]. Since this interval is entirely negative, it indicates that RF’s predictions are consistently higher than MLP’s predictions. RF appears to be the preferable model in this case because it has a higher correlation coefficient (0.9961), meaning its predictions are more accurate and aligned with the true values, even though its predictions are higher on average. The comparison between MLP and LWRF models reveals a negative t-statistic and a *p*-value < 0.05. The negative t-statistic suggests that LWRF’s predictions are, on average, higher than MLP’s predictions. The *p*-value shows that the difference between the predictions of LWRF and MLP is statistically significant. This means that the difference between the two models is real and not due to random chance. The 95% confidence interval for the mean difference between MLP’s and LWRF’s predictions is negative. This means that the true mean difference between MLP and LWRF predictions is likely to fall between −26.3263 and −10.2031, confirming that LWRF’s predictions are, on average, higher than MLP’s predictions. LWRF is the preferable model in this comparison because it has a higher correlation coefficient (0.9969), indicating that LWRF’s predictions are more accurate and aligned with the true values compared to MLP. In the MLP–XGBoost comparison, since the t-statistic is negative, the difference between the two models’ predictions shows that XGBoost tends to predict higher values compared to MLP. The *p*-value of 0.00023797 is less than 0.05, which means the observed difference between MLP’s and XGBoost’s predictions is statistically significant. The 95% confidence interval for the mean difference in predictions between MLP and XGBoost is negative [−25.9082, −8.9131]. This means that, on average, XGBoost’s predictions are higher than MLP’s predictions, and the difference is not close to zero. This further supports the claim that XGBoost performs better in terms of predicting higher values. XGBoost’s higher *CC* (0.99) compared to MLP’s *CC* (0.989) shows that XGBoost’s predictions are slightly more accurate and better aligned with the true values. Thus, based on the statistical significance and the higher correlation coefficient, XGBoost can be the preferable model in this comparison. In the MLP versus 1D-CNN comparison, the t-statistic of −2.378 indicates the CNN model’s predictions are, on average, higher than the MLP model’s predictions. *p*-value (0.0242) is less than 0.05, which means the observed difference in predictions between MLP and CNN is statistically significant. The negative confidence interval [−47.9652, −3.60878] suggests that CNN’s predictions are higher than MLP’s. MLP can be the preferable model in terms of accuracy, as indicated by its higher correlation coefficient (0.989), which suggests that MLP’s predictions are more aligned with the true values compared to CNN’s. In the MLP versus LSTM comparison, the results of the paired *t*-test indicate a statistically significant difference in the average predictions between the MLP and LSTM models. The negative t-statistic (−2.8813) reveals that the mean of the differences is negative. This implies that, on average, the predictions generated by the LSTM model are higher than those produced by the MLP model. The low *p*-value (0.0073788), which is below the conventional significance level of 0.05, further supports the rejection of the null hypothesis, confirming that the observed average difference is unlikely to have occurred by chance. The 95% confidence interval for the mean difference [−20.2701 to −3.43983], being entirely negative, provides further evidence that LSTM’s average predictions are significantly higher than MLP’s. Synthesizing the findings from both the paired *t*-test and the performance metrics, the MLP model demonstrates superior predictive performance compared to the LSTM model in this specific context. Despite the paired *t*-test indicating that LSTM’s predictions are, on average, higher, the significantly lower error metrics exhibited by the MLP suggest that its predictions are consistently closer to the actual values, leading to higher accuracy. Therefore, based on this comprehensive evaluation, the MLP model is the preferred choice for this particular problem. The last comparison for MLP is with the BiLSTM. The highly negative t-statistic (−5.6521) indicates a substantial and statistically significant difference in the average predictions between the MLP and BiLSTM models. The negative sign reveals that the mean of the differences is negative. This signifies that, on average, the predictions generated by the BiLSTM model are notably higher than those produced by the MLP model. The extremely low *p*-value (4.1531 × $10^{-6}$), far below the conventional significance level of 0.05, provides very strong evidence against the null hypothesis (no average difference between the models). This robustly confirms that the observed average difference is highly unlikely to have occurred due to random chance. The 95% confidence interval for the mean difference is entirely negative [−33.4811 to −15.6888] and relatively wide, further substantiating that BiLSTM’s average predictions are significantly and substantially higher than MLP’s. The range suggests the magnitude of this average difference. The results consistently point towards the superior performance of the MLP model over the BiLSTM model for this regression problem. While the paired *t*-test reveals that BiLSTM’s predictions are, on average, significantly higher than MLP’s, the performance metrics demonstrate that MLP achieves substantially lower prediction errors and a slightly better overall fit to the data. The higher average predictions of BiLSTM likely indicate a systematic upward bias. Therefore, based on the lower error rates and comparable or better correlation and explained variance, the MLP model is the preferred choice in this comparison.

The first comparison, which takes place in the SVR versus other models section, is the SVR–RF comparison. The highly negative t-statistic (−7.2688) indicates a very substantial and statistically significant difference in the average predictions between the SVR and RF models. The negative sign reveals that the mean of the differences is negative. This signifies that, on average, the predictions generated by the RF model are notably higher than those produced by the SVR model. The extremely low *p*-value (5.2696 × $10^{-8}$), far below the conventional significance level of 0.05, provides exceptionally strong evidence against the null hypothesis (no average difference between the models). This overwhelmingly confirms that the observed average difference is highly unlikely to have occurred due to random chance. The 95% confidence interval for the mean difference is entirely negative and relatively wide [−31.7941 to −17.831], strongly supporting that RF’s average predictions are significantly and substantially higher than SVR’s. The range indicates the considerable magnitude of this average difference. The results unequivocally demonstrate the superior performance of the Random Forest model over the SVR model for this regression problem. The paired *t*-test reveals that Random Forest’s predictions are, on average, significantly higher than SVR’s. Crucially, the performance metrics show that Random Forest achieves substantially lower prediction errors and a slightly better overall fit to the data, along with a marginally stronger linear correlation. The higher average predictions of Random Forest are associated with greater accuracy in this case. Therefore, the Random Forest model is the clearly preferred choice in this comparison. In the SVR–LWRF comparison, the t-statistic (−6.603) indicates a substantial and statistically significant difference in the average predictions between the SVR and LWRF models. The negative sign reveals that the mean of the differences (SVR predictions—LWRF predictions) is negative. This signifies that, on average, the predictions generated by the LWRF model are notably higher than those produced by the SVR model. The extremely low *p*-value (3.0906 × $10^{-7}$), far below the conventional significance level of 0.05, provides very strong evidence against the null hypothesis (no average difference between the models). This robustly confirms that the observed average difference is highly unlikely to have occurred due to random chance. The 95% confidence interval for the mean difference (SVR predictions—LWRF predictions) is estimated to lie between −35.7999 and −18.8672. This range implies 95% confidence that the true average difference between the predictions of SVR and LWRF in the broader population falls within these bounds. The fact that this interval is entirely negative further supports the conclusion that LWRF’s average predictions are significantly higher than SVR’s. The results consistently demonstrate the superior performance of the LWRF model over the SVR model for this regression problem. The paired *t*-test reveals that LWRF’s predictions are, on average, significantly higher than SVR’s. Importantly, the performance metrics show that LWRF achieves lower prediction errors and a better overall fit to the data, along with a slightly stronger linear correlation. The higher average predictions of LWRF are associated with greater accuracy in this case. Therefore, the LWRF model can be the clearly preferred choice in this comparison. In the SVR-LWRF comparison, the paired *t*-test reveals a highly statistically significant difference in the average predictions between the SVR and XGBoost models (t = −6.8409, *p* < 0.001). The 95% confidence interval for the mean difference (SVR predictions—XGBoost predictions) is [−34.396, −18.5629], which, being entirely negative and not containing zero, strongly supports this finding. This indicates that, on average, the XGBoost model generates notably higher predictions than the SVR model. Examining the performance metrics presents a more nuanced comparison. While both models exhibit strong correlations with the actual values (*CC*: SVR = 0.9942, XGBoost = 0.99) and explain a large proportion of the variance (*R*^2^: SVR = 0.9884, XGBoost = 0.9801), differences emerge in the error metrics. SVR demonstrates a lower *MAE* and *RAE*, suggesting smaller average absolute errors. Conversely, XGBoost achieves a lower *RMSE* and *RRSE*, indicating greater robustness to large errors. The choice between SVR and XGBoost might depend on the specific priorities of the application. If minimizing the average magnitude of errors is crucial, SVR might be preferred. If robustness to large errors is more important, XGBoost could be a better choice. Overall, the performance difference between the two models is relatively small based on these metrics, but the consistent statistical significance of the average prediction difference is noteworthy. For SVR—1D-CNN comparison, the 1D-CNN model exhibits significantly higher average predictions than the SVR model (t = −3.3932, *p* = 0.0020159, CI: [−55.8649, −13.8467]). In terms of predictive performance, however, the SVR model demonstrably outperforms the 1D-CNN, achieving much higher correlation and R-squared values, along with drastically lower average and root mean squared errors. Therefore, the SVR model can be the clearly preferred choice in this comparison. Upon examining the comparison between SVR and LSTM, the paired *t*-test (t = −6.0087 and *p* = 1.5551 × $10^{-6}$) demonstrates that LSTM’s predictions are, on average, significantly higher than SVR’s. The 95% confidence interval (−28.0458 to −13.8018) for the mean difference is entirely negative and relatively wide. This further reinforces that LSTM’s average predictions are significantly and substantially higher than SVR’s. However, the performance metrics overwhelmingly indicate that SVR outperforms LSTM in terms of prediction accuracy (lower *MAE* and *RMSE*), stronger linear relationship with the actual values (higher *CC*), and better overall fit to the data (higher *R*^2^). The higher average predictions of LSTM likely represent a systematic bias. Therefore, based on these results, SVR can be the preferred model for this regression problem. The final comparison involving SVR is conducted with the BiLSTM model. The paired *t*-test strongly rejects the null hypothesis of no average difference between the SVR and BiLSTM models (t = −8.3294, *p* < 0.001). The entirely negative 95% confidence interval for the mean difference (SVR predictions—BiLSTM predictions), spanning from −41.9172 to −25.3904, confirms that BiLSTM’s predictions are significantly and substantially higher on average than those of the SVR model. However, the performance metrics overwhelmingly demonstrate that SVR outperforms BiLSTM in terms of prediction accuracy (lower *MAE* and *RMSE*), stronger linear relationship with the actual values (higher *CC*), and better overall fit to the data (higher *R*^2^). The higher average predictions of BiLSTM likely represent a systematic upward bias and are associated with worse overall performance. Therefore, based on these results, SVR can be the preferred model for this regression problem.

Statistical comparison between RF and LWRF models reveals that RF tends to produce marginally lower prediction values than LWRF. The t-statistic of −1.9957 indicates that the mean difference in predictions (RF—LWRF) is slightly negative. The *p*-value of 0.0554 is slightly above the conventional threshold of 0.05, indicating that the difference between RF and LWRF is not statistically significant at the 5% level, although it is marginally close to significance. The confidence interval [−5.1045, 0.0625] includes zero, which supports the conclusion that the performance difference could be due to chance. There is no statistically significant difference between RF and LWRF based on their prediction outputs (*p* = 0.0554). In the RF-XGBoost comparison, the t-statistic (−0.71256) is close to zero, suggesting that there is no substantial difference in the average predictions between the Random Forest and XGBoost models. The *p*-value (0.4818) is well above the conventional significance level of 0.05. This indicates that rejection of the null hypothesis of no significant difference in the average predictions of the two models failed. The observed difference in average predictions is likely due to random chance. The 95% confidence interval for the mean difference (RF predictions—XGBoost predictions) includes zero [−6.4515 to 3.1176]. This also supports the conclusion that there is no statistically significant difference in the average predictions of the two models. However, the performance metrics consistently show that the RF model outperforms XGBoost in terms of prediction accuracy (lower *MAE* and *RMSE*), a slightly stronger linear relationship with the actual values (higher *CC*), and a better overall fit to the data (higher *R*^2^). Therefore, despite the lack of a significant difference in average predictions, the RF model can be the preferred choice. In the RF–1D-CNN comparison, the paired *t*-test (t = −0.7599, *p*-value = 0.4534, and CI = [−37.0711 to 16.9845]) indicates that there is no statistically significant difference in the average predictions between the RF and 1D-CNN models within this specific dataset. However, the performance metrics overwhelmingly demonstrate that the RF model significantly outperforms the 1D-CNN model in terms of prediction accuracy (much lower *MAE* and *RMSE*), a much stronger linear relationship with the actual values (higher *CC*), and a far better overall fit to the data (higher *R*^2^). Despite the lack of a significant difference in average predictions, the RF model can be the clearly preferred choice based on its vastly superior performance metrics. In the RF–LSTM comparison, although the paired *t*-test (t = 0.68993, *p* = 0.49572, 95% CI: [−7.639, 15.4165]) suggests no statistically significant difference in the average prediction levels of RF and LSTM for this data, a stark contrast emerges from the performance metrics. RF exhibits significantly better prediction accuracy, evidenced by its much lower *MAE* and *RMSE*, and demonstrates a stronger linear relationship and better variance explanation (higher *CC* and *R*^2^). Consequently, despite the *t*-test’s finding regarding average predictions, RF is the preferable model based on its substantially improved predictive capabilities. The final comparison involving RF is conducted with the BiLSTM model. The paired *t*-test (t = −2.1534, *p* = 0.03974, with a 95% confidence interval of [−17.2384, −0.444155]) shows that BiLSTM tends to predict higher values on average compared to RF. However, in terms of practical application, the RF model offers significantly better accuracy, as indicated by its considerably lower *MAE* and *RMSE*. Furthermore, its superior *CC* and *R*^2^ values suggest a more reliable and better-fitting model. Therefore, despite the difference in average prediction levels, RF can be the more suitable choice for this regression task.

In the LWRF–XGBoost comparison, LWRF tends to produce slightly higher predictions than XGBoost on average (the t-statistic of 0.3134 indicates a very small mean difference in prediction values between the two models). The *p*-value of 0.7562 is well above the 0.05 threshold, implying that the difference is not statistically significant. The confidence interval includes zero [−4.7196, 6.4277], further supporting the conclusion that the difference may be due to random variation. However, the performance metrics consistently demonstrate that the LWRF model outperforms XGBoost in terms of prediction accuracy (lower *MAE* and *RMSE*), a stronger linear relationship with the actual values (higher *CC*), and a better overall fit to the data (higher *R*^2^). Therefore, despite the lack of a significant difference in average predictions, the LWRF model can be the preferred choice based on its performance metrics. When analyzing the results of the LWRF–1D-CNN comparison, it is revealed that the t-statistic (−0.54077) is very close to zero, and the corresponding *p*-value (0.5928) is well above the common significance threshold of 0.05. It is not statistically evident for this specific dataset that the average predictions of LWRF and 1D-CNN are significantly different from each other. The 95% confidence interval for the mean difference (−35.9722 to 20.9277) contains zero. This reinforces the conclusion from the *p*-value that the observed differences in average predictions could easily be due to random variation. While the paired *t*-test suggests that there is not a statistically significant difference in the average prediction levels between LWRF and 1D-CNN on this particular dataset, the performance metrics paint a very clear picture of LWRF’s substantial superiority. The consistent and large differences in the error metrics strongly suggest that LWRF can be a far more reliable and accurate model for this regression task. According to LWRF–LSTM comparison, there is no significant difference in average predictions (t-statistic = 1.0232 and *p*-value = 0.3147). The 95% confidence interval for the mean difference [−6.40191 to 19.2214] contains zero. This reinforces the conclusion from the *p*-value that the observed differences in average predictions could easily be due to random variation. The interval spanning both negative and positive values suggests that sometimes LWRF’s predictions are higher and sometimes 1D-CNN’s are higher, with no consistent significant trend across the dataset. Based on this analysis, the LWRF model can be the clearly preferred choice due to its overwhelmingly superior performance across all relevant metrics, indicating much higher accuracy and a better fit to the data, regardless of the non-significant difference in average predictions. In the LWRF–BiLSTM comparison, the negative t-statistic (−1.3039) indicates that, on average, LWRF tends to produce lower prediction values than BiLSTM. However, the *p*-value (0.2025) is well above the 0.05 significance threshold, which means the observed difference in predictions is not statistically significant. The confidence interval contains zero [−16.2337, 3.5932], supporting the conclusion that the observed differences could be due to random variation. Despite the lack of statistical significance, the consistently better performance metrics suggest that LWRF may offer practical advantages over BiLSTM in this task.

Despite the paired *t*-test suggesting no statistically significant difference (t-statistic = −0.6452, *p*-value = 0.5239, and CI = [−34.9273, 18.1747]) in the average prediction levels between XGBoost and 1D-CNN for this specific dataset, the performance metrics reveal a clear and substantial advantage for XGBoost. The significantly higher *CC* and *R*^2^ values for XGBoost indicate a much better model capturing the underlying data patterns and explaining the variance. The dramatically lower *MAE* and *RMSE* values for XGBoost demonstrate a far greater level of prediction accuracy with considerably smaller errors compared to the 1D-CNN. In the XGBoost–LSTM comparison, the positive t-statistic (1.0433) indicates that, on average, XGBoost produces higher predictions than LSTM. However, the *p*-value of 0.3054 is much greater than the typical threshold of 0.05, meaning the difference is not statistically significant. The confidence interval contains zero [−5.3351, 16.4464], which confirms that the difference may be due to random chance. The observed differences are not sufficient to confidently assert model superiority based solely on statistical evidence. Despite the lack of statistical significance, XGBoost demonstrates clear practical advantages over LSTM in terms of lower prediction errors, higher correlation with actual values, and better explained variance. Comparison between the XGBoost and BiLSTM models reveals that there is a marginally non-significant difference in average predictions. The t-statistic of −1.9407 yields a *p*-value of 0.0621, which is slightly above the conventional significance level of 0.05. This suggests that while there is a tendency for the average predictions of BiLSTM to be higher than XGBoost’s, this difference is not statistically significant at the 0.05 level for this specific dataset. The 95% confidence interval for the mean difference [−14.735 to 0.386361] includes zero. This further supports the conclusion that it cannot be confidently stated that there is a significant average difference between the predictions of the two models based on this data. However, the performance metrics consistently and clearly demonstrate that XGBoost outperforms BiLSTM across all evaluated measures. XGBoost shows a stronger linear relationship with the actual values, explains more variance, and, most importantly, produces significantly lower prediction errors.

Upon examining the results of the comparison between CNN and LSTM, the t-statistic (1.5412) is not large enough in magnitude to yield a statistically significant *p*-value (0.13411, which is >0.05). This suggests that the average difference in predictions between the 1D-CNN and LSTM is not statistically different from zero. The 95% confidence interval for the mean difference is estimated to lie between −4.55613 and 32.4201. The fact that this interval encompasses both negative and positive values suggests that confidence about the direction of the true average difference between the two models is uncertain. In some instances, 1D-CNN’s predictions might be higher, while in others, LSTM’s might be, with the overall average difference not being statistically distinguishable from zero. However, the consistent and substantial advantages shown by the performance metrics strongly suggest that LSTM is a far more accurate and reliable model for this regression task since it has superior correlation and explained variance, significantly lower prediction errors, and more effective error reduction. Statistical comparison between 1D-CNN and BiLSTM reveals that 1D-CNN predictions are slightly higher than those of BiLSTM (t-statistic = 0.1067). However, the *p*-value of 0.9157 is far above the 0.05 threshold, indicating that this difference is not statistically significant. The confidence interval contains zero (from −21.83 to 24.23), confirming that the performance difference between 1D-CNN and BiLSTM may be due to random variation. In practice, BiLSTM provides substantially better predictive performance than 1D-CNN, despite the absence of statistically significant differences in their prediction outputs, since it has lower errors, higher correlation, and better fit.

The final statistical comparison is between LSTM and BiLSTM. According to the obtained results, the t-statistic of −2.7465 yields a *p*-value of 0.010241, which is below the common significance level of 0.05. This indicates that there is a statistically significant difference in the average predictions generated by the LSTM and BiLSTM models within this specific dataset. The 95% confidence interval for the mean difference (−22.2096 to −3.25043) is entirely negative. This confirms that the average prediction of BiLSTM is significantly higher than that of LSTM. The performance metrics present a mixed picture, with LSTM showing a slightly better overall fit and correlation, while BiLSTM has marginally lower absolute errors but higher squared errors.

## 4. Discussion

The results of this study highlight the significance of polymer-based phantom materials in proton therapy, especially in terms of LET behavior and Bragg curve characteristics [49,50]. When comparing the dose distributions and energy deposition patterns of polymers such as Parylene, Epoxy, Lexan, and Mylar across various proton energies, Mylar exhibited notably higher LET values [51]. This indicates increased biological effectiveness due to greater energy loss by protons within the material [52,53]. The position and shape of the Bragg peaks varied depending on the polymer type, revealing differences in the depth at which protons come to rest [54]. Such insights are critical for selecting optimal materials in phantom construction and ensuring the accuracy of treatment planning systems [55].

A recent study by Asuroglu [54] explored the use of various machine learning models to predict the Bragg peak position in proton therapy using polymeric materials. The algorithms assessed included Linear Regression (LR), SVR, kNN, RF, and XGBoost. According to the reported correlation coefficient (*CC*) values, LR exhibited the lowest performance (*CC* = 0.554), highlighting its inadequacy in capturing the complex, nonlinear nature of the problem. In contrast, RF (*CC* = 0.712), XGBoost (*CC* = 0.703), and kNN (*CC* = 0.675) demonstrated superior accuracy. The current study employed a partially overlapping set of algorithms—specifically SVR, kNN, RF, XGBoost—and also incorporated deep learning architectures including 1D-CNN, LSTM, and BiLSTM. Interestingly, while Asuroglu reported modest performance for SVR (*CC* = 0.596), the implementation of SVR yielded better accuracy, likely due to dataset-specific characteristics and kernel optimization. Moreover, the findings of this study are consistent with their observation that ensemble methods (RF, XGBoost) tend to outperform simpler models such as LR or basic SVR in the context of Bragg peak prediction. These cross-study results validate the choice of algorithms and further emphasize the importance of using nonlinear and ensemble-based models when dealing with energy deposition prediction tasks in complex biological or synthetic media.

Although the *RMSE* and *MAE* values reported in this study are relatively higher than those observed in some related works, such as Lee et al. [56], this discrepancy can be attributed to key differences in the dataset structure, prediction targets, and experimental design. Specifically, Lee et al. utilized a large and well-balanced dataset composed of over 5400 augmented quality assurance samples collected from a ten-year period, which likely enhanced the generalizability of their models. Furthermore, their prediction targets—proton range and spread-out Bragg peak width—are physical parameters measured with high precision using multilayer ionization chambers. In contrast, this study aims to predict Bragg peak locations from polymer-based LET profiles, which inherently exhibit higher variability due to complex interaction mechanisms within heterogeneous materials.

Additionally, the models used in their study are specifically optimized for low-error regression with narrow error bounds (e.g., within ±1 mm), which differs from the broader target range of this study. Consequently, in this study, although the *RMSE* values are higher, the correlation coefficients (*CC*) remain comparably strong, indicating that the models are effectively capturing the underlying trends in the data. These contextual factors suggest that direct comparisons based solely on *RMSE* or *MAE* may not adequately reflect model performance across different application settings. Therefore, the results in this study remain robust and relevant within the framework and objectives defined for this study.

In addition to the findings, a comparison with a recent study by Wang et al. [57] has been conducted, where deep learning-based protoacoustic signal denoising was applied to proton range verification. Although the focus of their study is on acoustic signal processing rather than regression-based prediction of Bragg peak locations from LET distributions, some shared insights can be drawn regarding model performance and methodological implications. For instance, their stacked autoencoder (SAE) approach yielded low range uncertainty (e.g., MEBP = 0.20 ± 3.44 mm for their high-accuracy detector using eight averaged signals), which highlights the effectiveness of deep learning in noisy data environments. Unlike their SAE-based method, this study evaluated a range of classical and deep regression algorithms, including SVR, MLP, and 1D-CNN, for direct Bragg peak prediction from LET profiles. In the present study, the *CC* metric was the central evaluation measure due to the high variability observed in absolute error metrics such as *RMSE* and *MAE*, variability that is likely due to the range and scale of Bragg peak targets in the dataset. Compared to the study by Wang et al., who did not report *CC* values explicitly but showed qualitative agreement in signal fidelity, the approach in this study emphasizes model generalization across multiple MeV levels and larger feature vectors. This is particularly significant given that the dataset contained broader variability in MeV inputs and Bragg peak targets. Future work could explore integrating denoising techniques prior to regression modeling, as carried out by Wang et al., to further enhance prediction accuracy under real-world measurement noise.

Furthermore, data normalization represents another critical aspect that requires discussion specifically for this study. Although feature normalization is commonly recommended for certain machine learning algorithms such as SVR or MLP, it was intentionally not applied in this study. This decision was based on the nature of the input features, which are physical measurements (MeV and LET values) that inherently carry meaningful absolute magnitudes. As emphasized by Garcia et al. [58], normalization methods like min–max scaling may be unsuitable when feature distributions are affected by outliers or when the minimum and maximum values are not well defined. Moreover, for certain models such as decision trees or ensemble methods, normalization is generally unnecessary due to their scale invariance. In the context of this research, it is aimed to preserve the original physical characteristics of the data, particularly since the input features reflect realistic energy and dose parameters. Therefore, omitting normalization was a deliberate choice to maintain the interpretability and integrity of the data.

The small size of the test set, particularly when evaluating relatively complex models such as 1D-CNN and BiLSTM, can introduce variability in the performance estimates. As a result, the reliability of these models’ evaluation may be affected due to increased variance. However, the primary goal in including these deep learning architectures was not only to assess raw predictive accuracy but also to explore their applicability and potential in modeling proton Bragg peak behavior using LET-based feature representations.

Deep learning models, particularly LSTM and BiLSTM, are known to require large volumes of data to achieve stable generalization and to prevent overfitting. In our study, the training dataset consists of only 90 samples (30 per polymer type), which is a relatively small size for training neural networks with hundreds or thousands of parameters. Despite applying dropout and regularization techniques (e.g., spatial dropout in CNN, recurrent dropout in LSTM), the model complexity likely exceeded the learning capacity that the data could support. This is consistent with prior studies indicating that deep architectures tend to overfit or under-generalize when sample sizes are limited.

Although the 200 LET values per sample form a numerical sequence, they represent spatially resolved physical measurements rather than temporally evolving signals. As such, the assumption of temporal dependencies, a core strength of LSTM/BiLSTM, is likely not fully met. The weak sequential structure may have diminished the advantage of recurrent architectures. Moreover, local dependencies in LET profiles might not be strong or consistent enough to benefit from the convolutional inductive bias of 1D-CNN.

The deep models were trained on CPU-based hardware and tuned within restricted parameter spaces to ensure comparability and training feasibility. This hardware constraint also limited the extent of hyperparameter exploration, especially for LSTM and BiLSTM, which are computationally expensive and sensitive to architectural depth and learning rate tuning. Thus, under-optimization may have occurred despite grid search application.

Several strategies are proposed to address these issues in future work:

Generating synthetic LET profiles using noise models or generative frameworks could increase dataset size and diversity. Pretraining on a larger simulated or clinical LET dataset followed by fine-tuning on the specific polymer subset could improve generalization. Combining CNN/LSTM layers with attention mechanisms or physics-informed priors may help capture spatial relevance without relying strictly on temporal structure. Rather than using raw LET profiles, extracting physical descriptors (e.g., peak width, gradient, area under curve) may provide models with more informative inputs.

While the use of an entirely unseen polymer (Epoxy) for independent testing strengthens the internal validity of our generalization assessment, the relatively small number of test instances inherently restricts the statistical power of the evaluation. With only 30 samples, variance in model performance may be driven in part by individual outliers or material-specific patterns that are not representative of broader trends.

Moreover, no external datasets or clinical validation were incorporated in this study, primarily due to the absence of publicly available, standardized LET datasets from independent sources. This limitation affects the external validity and real-world generalizability of our findings, particularly in clinical or cross-institutional contexts where different polymer grades, experimental settings, or beam configurations may be present.

To address this concern, the following steps are proposed for future work:

Future studies should include a larger number of samples per polymer and consider incorporating additional materials not present in the current training or test sets. Acquiring LET and Bragg peak data from different research centers, hospitals, or simulation set-ups would enable broader model generalization assessment and promote reproducibility. Integrating external datasets or benchmarking against real-world measurements (e.g., from phantom experiments) would enhance the clinical relevance of the findings. With a larger and more diverse dataset, further statistical techniques such as bootstrapping, permutation testing, or nested cross-validation could be employed to improve confidence intervals and mitigate sample bias.

While the predictive models developed in this study demonstrate promising performance on an unseen material type (Epoxy), it is important to note that the generalizability of the models across broader polymer classes remains limited due to the absence of a second independent validation dataset. The test set design, which includes only Epoxy samples, was intended to simulate a material-level holdout scenario; however, this restricts the conclusions that can be drawn regarding the overall robustness and transferability of the models. To fully assess the applicability of the proposed workflow in real-world settings, future studies should include independent evaluation on additional polymer types not used during model training. This limitation is explicitly acknowledged and presented as an important direction for future research.

The modeling results obtained through artificial intelligence revealed that algorithms like RF, LWRF, and SVR achieved superior performance with high correlation coefficients and low error metrics. These findings demonstrate that AI-based models can predict LET profiles with high accuracy, offering a reliable alternative to labor-intensive experiments. Furthermore, the statistical comparison of different AI algorithms provided a comprehensive perspective on model suitability for specific applications. Overall, this study proposes a holistic framework for material selection in proton therapy, integrating both physical characterization and advanced data-driven modeling techniques.

Each algorithm was compared with the other eight algorithms to determine whether the differences were statistically significant. In these comparisons, the primary focus is not only on identifying which algorithm yields higher values in terms of the *CC* and consequently the *R*^2^ metrics but also on determining whether the differences in the predictive performance between the two compared algorithms are statistically significant. Table 5 presents the number of times each algorithm outperformed the others in terms of the *CC* and *R*^2^ metrics, along with how many of these instances were statistically significant. A paired *t*-test was used to test for significant differences in predictions, while model superiority was assessed based on the correlation coefficient (*CC*). In cases of significant difference (*p* < 0.05), the model with the higher *CC* was considered superior. Detailed t-statistics and confidence intervals are provided in Table 3 and the text following Table 3.

**Figure 5:**
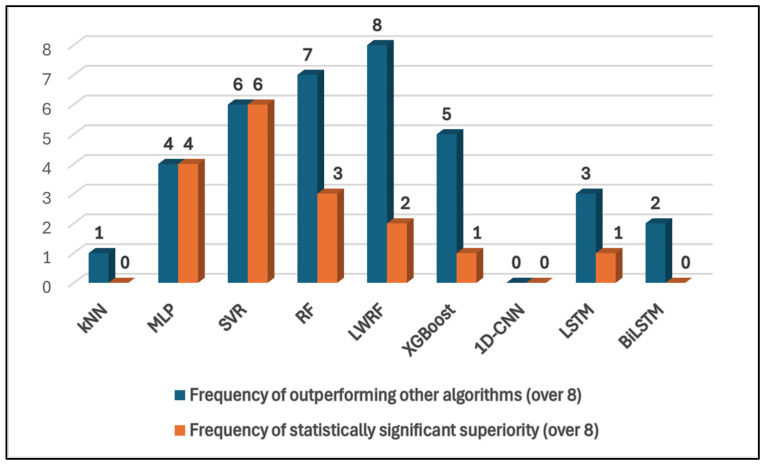
Pairwise comparison of nine models in terms of performance and statistical significance.

According to Table 5, SVR emerges as the most statistically robust model. It outperformed six models, all with statistically significant results, yielding the highest success rate of 75%. Notably, it demonstrated superiority over a diverse set of models, underlining its consistent and statistically validated effectiveness. MLP achieved a 50% success rate by outperforming four models, all with statistically significant differences. This indicates strong, consistent performance, particularly against kNN, 1D-CNN, LSTM, and BiLSTM, models that are generally ranked lower. RF shows high raw competitiveness by outperforming seven models; however, only three of these victories were statistically significant (success rate: 37.5%). While RF’s superiority against MLP, SVR, and BiLSTM is notable, the lack of consistent statistical backing weakens its overall reliability. LWRF demonstrates the highest raw frequency (8/8) of outperforming others, suggesting strong model capability. However, only two comparisons were statistically significant, limiting its success rate to 25%. This implies that its dominance may not be statistically reliable in most cases. XGBoost outperformed five models, but only its superiority over MLP was statistically significant, resulting in a 12.5% success rate. LSTM outperformed three models (including BiLSTM) with only one significant result, yielding the same success rate (12.5%) as XGBoost. kNN, 1D-CNN, and BiLSTM did not show any statistically significant superiority and had limited or no model-level wins, indicating poor performance and low statistical competitiveness (Figure 5).

The analysis clearly highlights SVR as the most effective and statistically reliable model in this evaluation. MLP also performed competitively with solid statistical support. Although RF and LWRF exhibited high raw outperforming frequencies, their statistical reliability is weaker. The performance of XGBoost, LSTM, and especially kNN, 1D-CNN, and BiLSTM remains limited under statistical scrutiny. This table supports a performance hierarchy where statistical significance serves as a crucial filter for validating algorithmic superiority, not just raw outperforming frequency.

While SVR demonstrated slightly superior performance to MLP in terms of correlation coefficient (*CC*), other important aspects must be considered for real-world deployment. One such factor is model interpretability. SVR models, especially with linear or simple kernel functions, are often more interpretable, allowing practitioners to better understand the relationship between input features and predictions. In contrast, MLPs, due to their multi-layered and non-linear structure, are frequently regarded as black boxes, which may hinder their adoption in domains where model transparency is critical. Another practical consideration is computational efficiency. SVR typically requires fewer hyperparameters and converges rapidly, making it well-suited for scenarios with computational constraints or where rapid retraining is necessary. MLPs, on the other hand, may involve longer training times and higher computational costs, especially as the network depth and size increase. Therefore, while SVR yielded marginally better predictive accuracy, the final choice between SVR and MLP should also weigh these real-world factors. If model transparency, interpretability, and rapid deployment are primary concerns, SVR may be preferable. Conversely, MLPs might be more appropriate in contexts where the flexibility to model highly non-linear relationships is essential and computational resources are not a limiting factor.

Statistical robustness in this context refers to the consistency of the SVR model’s predictions with respect to the true trend in the data. SVR achieved a high correlation coefficient (*CC* = 0.9942), which indicates that its predictions track the direction and pattern of the ground truth values quite closely. This strong linear relationship often results in low *p*-values when compared with other models in paired *t*-tests. However, correlation-based metrics such as *CC* do not fully reflect pointwise accuracy. It is entirely possible for a model to be highly correlated with the target while still exhibiting larger individual deviations, especially around outliers or in edge regions of the data distribution. SVR may have produced systematic prediction errors that are small in variance but slightly biased, leading to strong correlation but slightly higher *MAE* and *RMSE*. In contrast, although models such as LWRF or RF may exhibit higher variance in their predictions, they achieved better pointwise accuracy and therefore yielded lower error metrics. This means that SVR’s predictions are statistically stable and directionally aligned, but not necessarily the closest in magnitude to the true Bragg peak values. Thus, SVR’s advantage in statistical tests likely stems from its stable behavior over the test set rather than its pointwise optimality.

To address the risk of overfitting, regularization strategies were incorporated into the design of both the MLP and BiLSTM models. The MLP architecture included only a single hidden layer, after which a dropout layer with a rate of 0.3 was applied to promote generalization by randomly deactivating a subset of neurons during training. Similarly, dropout was used after each recurrent layer in the BiLSTM network.

Throughout model training, validation loss was continuously monitored. Early stopping was utilized based on validation performance, halting training if no improvement was observed for five consecutive epochs. Notably, the MLP, despite its simple architecture, demonstrated not only lower error rates on the test set but also a stable validation loss curve with no indication of severe overfitting. The BiLSTM, while achieving higher training accuracy, exhibited a slightly larger gap between training and validation performance, suggesting a greater propensity for overfitting despite the use of dropout. These findings underscore the importance of regularization and validation monitoring when evaluating deep learning models for regression tasks.

In proton therapy, the clinical efficacy hinges critically on the accurate placement of the Bragg peak, which is the point at which protons deposit the maximum dose of radiation just before stopping. Accurate localization of this peak ensures maximal tumor control while sparing adjacent healthy tissues. AI-driven prediction of Bragg peak positions introduces a significant advancement in this domain.

AI models, trained on multimodal imaging (e.g., CT, MRI), can learn patient-specific anatomical and tissue heterogeneity patterns to predict proton stopping power ratios (SPRs) with higher accuracy than conventional heuristic models. As a result:

- Treatment planning accuracy is improved by reducing range uncertainties.
- Adaptive radiotherapy becomes feasible through real-time Bragg peak re-estimation, accommodating changes in tumor volume, patient anatomy, or positioning.
- Dose conformality is enhanced, leading to reduced toxicity and better clinical outcomes, particularly in anatomically complex regions such as the head and neck.

Despite its promise, integration of AI-predicted Bragg peaks into clinical practice faces several critical challenges:

- AI-based medical systems require rigorous validation and approval from regulatory agencies such as the FDA or EMA. The lack of standardized pathways for adaptive AI tools further complicates this process.
- Models trained on limited or homogeneous datasets may perform poorly on unseen populations due to domain shift (e.g., imaging protocol variations, ethnic anatomical differences). This limits scalability across institutions.
- AI systems often operate as “black boxes.” Clinicians may be reluctant to trust outputs they cannot interpret, especially when patient safety is at stake.
- Current treatment planning systems are not natively compatible with AI modules, and their integration requires substantial technical and institutional investment.
- Issues around accountability in decision-making (AI vs. physician), data privacy, and model bias remain unresolved in many jurisdictions.

To overcome these barriers and facilitate clinical translation, the following strategies are recommended:

- Combine AI predictions with physics-based dose calculation methods (e.g., Monte Carlo simulations) to ensure reliability while leveraging AI’s speed and adaptability.
- Implement interpretable models (e.g., attention maps, SHAP values) to provide clinicians with intuitive explanations for AI-driven Bragg peak predictions.
- Use federated learning to train models across diverse institutions without sharing sensitive data, thereby improving generalizability and privacy compliance.
- Establish protocols for the clinical use of AI in proton therapy, including validation standards, performance metrics, and fail-safe mechanisms.
- Offer targeted training for oncologists, dosimetrists, and physicists to build competence and confidence in AI-assisted planning tools.

## 5. Conclusions

The key contributions and findings of this study are summarized as follows:

- A total of nine machine learning and deep learning models were applied to predict Bragg peak positions in Epoxy polymer based on LET and MeV features.
- To the best of our knowledge, this is the first study to apply the LWRF algorithm for Bragg peak prediction.
- The models were trained on data from three distinct polymer materials (Mylar, Lexan, and Parylene) and tested on a completely unseen material (Epoxy), enabling generalization assessment.
- Based on the *CC* metric, LWRF, RF, and SVR were the top three performing models, respectively, demonstrating the highest predictive correlations.
- Among all the algorithms evaluated, SVR produced statistically significant results and demonstrated the most consistent performance differences.
- This comparative framework can inform future applications of AI in proton therapy and energy deposition modeling.

The motivation behind this study was to develop a machine learning-based framework for accurately predicting Bragg peak locations in polymeric materials under proton irradiation, with the ultimate aim of improving computational tools used in proton therapy research. The models were trained on a high-dimensional dataset incorporating energy and LET profiles, and their prediction accuracies were systematically compared.

This study presents an approach to predicting the penetration depth of subatomic particles within Epoxy materials by leveraging models trained on data obtained from Parylene, Lexan, and Mylar. This indirect methodology, utilizing structurally and compositionally distinct polymers for model training, offers a unique pathway to circumvent the often resource-intensive and experimentally challenging process of directly measuring particle penetration depths in Epoxy across a spectrum of MeV values.

The most successful models (RF, LWRF, and SVR) highlight the importance of modeling complex, non-linear behavior in radiation-based problems. The workflow proposed in this study can be adapted to predict other proton therapy parameters, such as dose distribution or proton range, although this would require incorporating additional physical and anatomical data.

As for limitations, models such as kNN and BiLSTM performed poorly due to their limited capacity in capturing deep, non-linear dependencies in the input space. These limitations suggest that future studies should consider feature selection techniques or hybrid models to improve generalization. Although 1D-CNN architectures are generally effective for extracting local patterns in sequential data, the relatively weak performance of the 1D-CNN model in this study may be attributed to two main factors. First, the high dimensionality of the input vectors (200 LET values per sample) combined with a lack of spatial locality in the data may limit the convolutional filters’ ability to extract meaningful features. Unlike image or time-series data, the ordering of LET features in this case does not represent a naturally correlated structure, which diminishes the benefits of local receptive fields. Second, 1D-CNNs rely heavily on hierarchical abstraction through depth; the model might have been insufficiently deep or expressive to capture the complex non-linearities present in the energy–LET–Bragg peak relationship. This suggests that future studies using convolutional approaches may benefit from employing hybrid models or attention mechanisms that do not rely on strict locality assumptions.

The outcomes of this study contribute a replicable workflow that can be directly applied to other biomedical or radiological prediction tasks. In future research, it is aimed to extend this pipeline to incorporate Monte Carlo simulated dose maps and anatomical CT data, enabling a more holistic prediction framework in clinical settings.

The potential utility of this research lies in several key areas. Firstly, it introduces a cost-effective and time-efficient alternative to direct experimental measurements. Characterizing particle interactions with materials, particularly at varying energy levels, typically requires specialized equipment and meticulous experimental design. By demonstrating the feasibility of training predictive models on readily available data from other polymeric materials, this work could significantly reduce the experimental burden associated with material characterization for specific applications like Epoxy.

Secondly, the study holds promise for accelerating material screening and selection processes. Epoxy resins are widely employed in diverse fields, including electronics, aerospace, adhesives, and coatings, due to their excellent mechanical, electrical, and chemical properties. The ability to rapidly and accurately predict particle penetration depths without extensive direct experimentation could expedite the identification of optimal Epoxy formulations for applications where radiation shielding or interaction with high-energy particles is a critical factor. This is particularly relevant in environments such as nuclear facilities, medical radiation therapy, and space applications.

Furthermore, the methodology could contribute to a deeper understanding of the underlying physical principles governing particle–matter interactions. While the training data originate from Parylene, Lexan, and Mylar, the successful prediction of penetration depths in Epoxy suggests the existence of transferable patterns or relationships between the material properties and particle interaction mechanisms across different polymer types. Further investigation into the specific features learned by the models could yield valuable insights into the fundamental factors influencing particle penetration.

The findings of this study also have implications for the development and validation of computational models used in material science and nuclear engineering. By providing a benchmark against which simulations can be compared, this research can contribute to the refinement and increased accuracy of theoretical models predicting particle transport in complex materials.

In conclusion, this research offers a significant contribution to the field by presenting an innovative, indirect approach to predicting subatomic particle penetration in Epoxy. Its potential benefits include reduced experimental costs and time, accelerated material screening, enhanced understanding of particle–matter interactions, and improved validation of computational models. This work holds considerable promise for advancing material science research and informing material selection in various technological applications where particle interaction is a crucial design parameter.

To further validate the robustness and generalizability of this approach, future work could explore its applicability to a wider range of both training and target materials. Investigating the predictive power of models trained on a more diverse set of polymers (e.g., polyethylene, polypropylene, and nylon) for predicting penetration in other thermoset or thermoplastic materials would be a valuable extension. Additionally, exploring the model’s performance in predicting the penetration depths of different types of subatomic particles (e.g., alpha particles, neutrons) with varying energy ranges would broaden the scope and impact of this research.

The proposed machine learning-based workflow developed in this study can be extended to predict other key parameters of proton therapy, such as dose distribution, proton range, LET profiles, or even biological endpoints like relative biological effectiveness (RBE). The core advantage of the workflow lies in its modularity: by simply replacing the output variable and adjusting the input features accordingly, the same structure of data preparation, model selection, training, and evaluation can be applied to different predictive targets. For example, predicting dose distribution would involve using the same energy and LET vectors as inputs but training the models on voxel-wise dose values obtained through simulations or measurements. Similarly, if the proton range is the parameter of interest, the workflow could incorporate additional anatomical or material-specific descriptors alongside energy levels. Likewise, RBE predictions would require biological input features such as tissue type, oxygenation status, or experimental cell survival data, but the learning pipeline would remain consistent.

However, it should be noted that such extensions may require careful adjustments. Each target parameter may differ in terms of data complexity, scale, and sensitivity to input noise. Some parameters may demand multi-output regression or classification models, while others might benefit from physics-informed machine learning strategies or hybrid modeling approaches. Despite these considerations, the core workflow presented in this study serves as a flexible foundation for such expansions. Future work will focus on integrating additional parameters into the current pipeline to create a more comprehensive and clinically applicable prediction tool for proton therapy planning and optimization.

## Figures and Tables

**Table 1:** Performance of regression models in predicting Bragg peak values based on standard evaluation metrics (**bold** values represent the highest performance in the respective metric).

Model	*CC*	*MAE*	*RMSE*	*RAE*	*RRSE*	*R^2^*
**kNN**	0.9438	35.7663	50.6987	30.0673	36.6534	0.8908
**MLP**	0.9890	16.6705	23.3329	14.0142	16.8689	0.9781
**SVR**	0.9942	16.5358	24.8297	13.9010	17.9510	0.9884
**RF**	0.9961	**12.3161**	**15.8223**	**10.3536**	**11.4389**	0.9922
**LWRF**	**0.9969**	12.8080	16.9999	10.7672	12.2903	**0.9938**
**XGBoost**	0.9900	17.5839	22.7028	14.6842	16.4536	0.9801
**1D-CNN**	0.8735	66.0972	76.5860	55.1975	55.5048	0.7630
**LSTM**	0.9833	27.9468	33.7693	23.3382	24.4740	0.9669
**BiLSTM**	0.9763	27.7747	35.1835	23.1945	25.4988	0.9532

**Table 2:** Evaluation of estimation bias across models based on paired *t*-test results (**bold** *p*-values, *p* < 0.05, indicate statistical significance).

Model	t-Statistic	*p*-Value (<0.05)
kNN	−1.9112	0.0659
MLP	1.4236	0.1652
SVR	4.0962	**0.0003**
RF	−4.2348	**0.0002**
LWRF	−5.6459	**4.2253 × 10^−6^**
XGBoost	−3.1444	**0.0038**
1D-CNN	−1.4431	0.1597
LSTM	−0.9542	0.3479
BiLSTM	−3.3594	**0.0022**

**Table 3:** Statistical significance test results comparing the performance of machine learning and deep learning models for Bragg peak prediction (**bold** *p*-values indicate statistical significance).

	t-Statistic								
	p-Value (<0.05)								
	95% Confidence Interval (CI)								
Models	kNN	MLP	SVR	RF	LWRF	XGBoost	1D-CNN	LSTM	BiLSTM
**kNN**		2.5037	3.4631	0.8831	0.5762	0.668	−0.1823	1.177	−0.1887
		**0.0182**	**0.0017**	0.3845	0.5689	0.5094	0.8566	0.2488	0.8516
		[4.1968, 41.6438]	[13.0973, 50.881]	[−9.4446, 23.7978]	[−11.8694, 21.1806]	[−11.3589, 22.3783]	[−35.0216, 29.2883]	[−8.16207, 30.2927]	[−19.7046, 16.3752]
**MLP**			3.5596	−4.3858	−4.6337	−4.1905	−2.3781	−2.8813	−5.6521
			**0.0013**	**1.3914 ×** $10^{-4}$	**7.0126 ×** $10^{-5}$	**2.3797 ×** $10^{-4}$	**0.0242**	**0.0074**	**4.1531 ×** $10^{-6}$
			[3.85813, 14.2795]	[−23.0854, −8.40197]	[−26.3263, −10.2031]	[−25.9082, −8.9131]	[−47.9652, −3.60878]	[−20.2701, −3.4398]	[−33.4811, −15.6888]
**SVR**				−7.2688	−6.603	−6.8409	−3.3932	−6.0087	−8.3294
				**5.2696 ×** $10^{-8}$	**3.0906 ×** $10^{-7}$	**1.6334 ×** $10^{-7}$	**2.0158 ×** $10^{-3}$	**1.5551 ×** $10^{-6}$	**3.5059 ×** $10^{-9}$
				[−31.7941, −17.831]	[−35.7999, −18.8672]	[−34.396, −18.5629]	[−55.8649, −13.8467]	[−28.0458, −13.8018]	[−41.9172, −25.3904
**RF**					−1.9957	−0.7126	−0.7599	0.6899	−2.1534
					0.0554	0.4818	0.4534	0.4957	**0.0397**
					[−5.1045, 0.0625]	[−6.4515, 3.1176]	[−37.0711, 16.9845]	[−7.639, 15.4165]	[−17.2384, −0.4442]
**LWRF**						0.3134	−0.5408	1.0232	−1.3039
						0.7562	0.5928	0.3147	0.2025
						[−4.7196, 6.4277]	[−35.9722, 20.9277]	[−6.40191, 19.2214]	[−16.2337, 3.5932]
**XGBoost**							−0.6452	1.0433	−1.9407
							0.5239	0.3054	0.0621
							[−34.9273, 18.1747]	[−5.3351, 16.4464]	[−14.735, 0.386361]
**1D-CNN**								1.5412	0.1067
								0.1341	0.9157
								[−4.55613, 32.4201]	[−21.8312, 24.2321]
**LSTM**									−2.7465
									**0.0102**
									[−22.2096, −3.25043]
**BiLSTM**									

**Table 4:** Comparison of models according to the *CC* metric. An upward (↑) or leftward (←) arrow indicates that the algorithm to which the arrow points exhibits higher performance in terms of the *CC* metric compared to the other algorithm (**bold** arrows solely serve to highlight statistically significant differences between the outcomes of model pairs).

Models	kNN	MLP	SVR	RF	LWRF	XGBoost	1D-CNN	LSTM	BiLSTM
kNN		↑	↑	↑	↑	↑	←	↑	↑
MLP			↑	↑	↑	↑	←	←	←
SVR				↑	↑	←	←	←	←
RF					↑	←	←	←	←
LWRF						←	←	←	←
XGBoost							←	←	←
1D-CNN								↑	↑
LSTM									←
BiLSTM									

**Table 5:** Comparison of the models in terms of statistical significance (**bold** values indicate the number of times each algorithm demonstrated statistically significant difference over others based on the paired *t*-test (*p* < 0.05)).

Model	Frequency of Outperforming Other Algorithms (Over 8)	Frequency of Statistically Significant Superiority (Over 8)	Success Rate (%)	Outperformed Models
kNN	1	0	0	N/A
MLP	**4**	**4**	50	kNN, 1D-CNN, LSTM, BiLSTM
SVR	**6**	**6**	**75**	kNN, MLP, XGBoost, 1D-CNN, LSTM, BiLSTM
RF	7	**3**	37.5	MLP, SVR, BiLSTM
LWRF	8	**2**	25	MLP, SVR
XGBoost	5	**1**	12.5	MLP
1D-CNN	0	0	0	N/A
LSTM	3	**1**	12.5	BiLSTM
BiLSTM	2	0	0	N/A

## Data Availability

The data presented in this study are openly available in [Google Drive] at [https://tinyurl.com/2yb3fra6] (accessed on 5 May 2025), reference number [21].
